# Estimates of incidence, prevalence, mortality, and disability‐adjusted life years of lung cancer in Iran, 1990–2019: A systematic analysis from the global burden of disease study 2019

**DOI:** 10.1002/cam4.4792

**Published:** 2022-06-13

**Authors:** Zahra Shokri Varniab, Yeganeh Sharifnejad Tehrani, Ashkan Pourabhari Langroudi, Sina Azadnajafabad, Negar Rezaei, Mohammad‐Mahdi Rashidi, Zahra Esfahani, Mohammad‐Reza Malekpour, Erfan Ghasemi, Azin Ghamari, Arezou Dilmaghani‐Marand, Sahar Mohammadi Fateh, Alireza Namazi Shabestari, Bagher Larijani, Farshad Farzadfar

**Affiliations:** ^1^ Non‐Communicable Diseases Research Center, Endocrinology and Metabolism Population Sciences Institute Tehran University of Medical Sciences Tehran Iran; ^2^ Endocrinology and Metabolism Research Center, Endocrinology and Metabolism Clinical Sciences Institute Tehran University of Medical Sciences Tehran Iran; ^3^ Department of Biostatistics University of Social Welfare and Rehabilitation Sciences Tehran Iran; ^4^ Department of Geriatric Medicine, School of Medicine Tehran University of Medical Sciences Tehran Iran

**Keywords:** global burden of disease, Iran, lung cancer

## Abstract

**Background:**

Lung cancer is one of the leading cancers, with a high burden worldwide. As a developing country, Iran is facing with population growth, widespread tobacco use, demographic and epidemiologic changes, and environmental exposures, which lead to cancers becoming a severe concern of public health in Iran. We aimed to examine the burden of lung cancer and its risk factors in Iran.

**Methods:**

We utilized the Global Burden of Disease 2019 data and analyzed the total burden of the lung cancer and seven related risk factors by sex, age at national and sub‐national levels from 1990 to 2019.

**Results:**

The lung cancer age‐standardized death rate increased from 11.8 (95% Uncertainty Interval: 9.7–14.4) to 12.9 (11.9–13.9) per 100,000 between 1990 and 2019. This increase was among women from 5 (4.2–7.1) to 8 (7.2–8.8) per 100,000; in contrast, there was a decline among men from 18.5 (14.8–22.6) to 17.8 (16.2–19.4) per 100,000. The burden of lung cancer is concentrated in the advanced age groups. Smoking with 53.5% of total attributable deaths (51.0%–55.9%) was the leading risk factor. At the provincial level, there was a wide range between the lowest and highest, from 8.3 (7.0–10.0) to 19.1 (16.4–22.0) per 100,000 population in the incidence rate and from 8.7 (7.3–10.3) to 20.6 (17.7–24.0) per 100,000 population in mortality rate, respectively in Tehran and West Azerbaijan provinces in 2019.

**Conclusion:**

The increasing trend of lung cancer burden among the entire Iranian population, the inter‐provincial disparities, and the significant rise in burden of this cancer in women necessitate the urgent implementation and development of policies to prevent and manage lung cancer burden and strategies to reduce exposure to risk factors.

## Graphical abstract

Age‐standardized incidence, DALYs, and death rate in females were lower than males but had a remarkable increase. The study showed that the highest incidence and DALYs rate of LC were in the age group >70. Smoking was the first leading risk factor among the overall population, men, and all provinces of Iran.

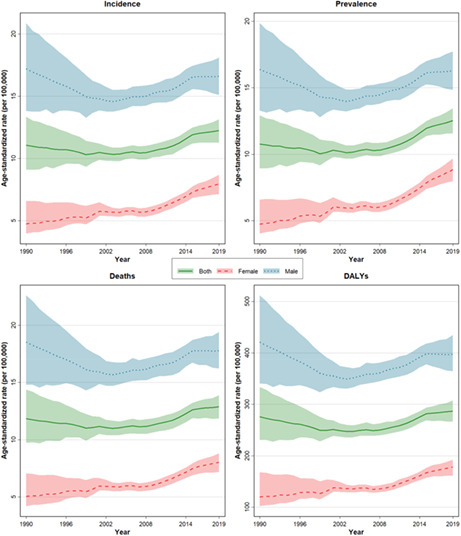

## 1 INTRODUCTION

Lung cancer (LC) was one of the top causes of death worldwide in 2019. Incidence cases of LC were 2.26 million (95% uncertainty interval [UI] 2.07–2.45), deaths were 2.04 million (1.88–2.19), and disability‐adjusted life year (DALYs) were 45.9 million (42.3–49.3) globally in 2019.^1^ Also, according to GLOBOCAN study, LC is the most common cancer in death and incidence among men in Iran.^2^ In Iran, cancer is *the third most significant cause of death* after heart diseases and accidents.^3^ A previous study indicated the ASIR for lung cancer has increased from 1.3 (0.7–1.9) in 1990 to 8.9 (7.3–10.5) in 2016. However, it showed an almost stable trend in mortality rate during the 27‐year period.^4^ LC is less frequent in Iran than western countries; in addition, cigarette consumption, especially among females, is *less prevalent* in Iran than other countries, which is the leading cause of the lower incidence of LC in Iran.^5^ There are *inter*‐*provincial disparities* in Iran. Many factors contribute to this difference, including the financial barriers in impeding access to health services and their quality, and disproportion in the screening, diagnosis, and treatment of NCD in the country's different provinces.^6^ A previous study has shown that the highest frequency of LC is in the western provinces of Iran, like Kurdistan and West Azerbaijan.^7^

LC is a multifactorial disease and has several most‐studied renowned risk factors, such as tobacco consumption, second‐hand smoking, previous radiation therapy to the lungs, genetic susceptibility, environmental and occupational exposures such as radon, asbestos, air pollution, and arsenic.^8^ Cigarette smoking is the single most significant and preventable risk factor of LC.^9^, ^10^ The difference in LC epidemiology worldwide indicates the quantity of tobacco consumption and the implementation of tobacco control strategies.^11^

As a developing country, Iran is susceptible to population growth, widespread tobacco use, facing demographic transition, and environmental exposures, which cause those non‐communicable diseases (NCDs) to become a severe concern of public health in Iran, especially cancers.^5^, ^6^, ^12^ The most common form of tobacco used in Iran is cigarette smoking; though, the most predominant form in women is hookah.^12^ According to the Iran STEPwise approach to surveillance of NCD risk factors (STEPS) survey 2016, 9.6% of participants are current cigarette smokers, while hookah is used by 0.2% of the population. Regardless of location, the prevalence of secondhand smoking was 31.5% among the adult population.^13^ A study showed that ambient particulate matter concentrations in Iran exceeded the World Health Organization (WHO) guideline values as an occupational risk. Approximately 668 lung cancer deaths occurred in Iran in 2016 as a consequence of long‐term exposure to particulate matter.^14^ Moreover, one of the major air pollutants is sulfur dioxide (SO_2_). A study indicated that the SO_2_ concentration in Iran increased from 22.00 parts ppb in 1990 to 27.81 ppb in 2015.^15^

The current study aimed to describe the trends of mortality, DALYs, and incidence in LC by sex, age groups, and related risk factors in Iran during the years of 1990–2019 at national and subnational scales following the Global Burden of Disease (GBD) study 2019. Moreover, providing valid and trusted data of patterns and changing trends of LC in Iran and provinces could help policymakers make effective policies and allocate resources appropriately which are critical steps in controlling the LC burden.

## 2 METHODS

### 2.1 Data source

### 2.2 GBD estimation framework

Using data from the GBD study 2019, data of the LC burden were analyzed in the current study and the trends of incidence, DALYs, and death of LC and the responsible risk factors were depicted by age and sex in Iran and 31 provinces of Iran. The GBD 2019 study was a comprehensive and systematic global research program to estimate the burden of 369 diseases and injuries and 87 risk factors in 204 countries and territories by sex and age since 1990. The GBD study evaluates different parameters of global health and burden of diseases, including incidence, prevalence, mortality, years of life lost (YLLs), years lived with disability (YLDs), healthy life expectancy (HALE), and DALYs (DALYs is defined as the sum of YLLs and YLDs) by age, sex, year, and location, and socio‐demographic index (SDI) (SDI is a measure of the development of a country based on fertility, average income, and years of education).^16^ Methodological details of the GBD 2019 study have been described in previous studies.^17^, ^18^, ^19^, ^20^ All results are available on the GBD compare tool,^21^ and all data about the burden of LC in the current study were available on the Global Health Data Exchange website.^22^ CODEm and CoDCorrect models have been used for estimations in the GBD study.^23^, ^24^ The CODEm (Cause of Death Ensemble model) estimates death with available and many different covariates such as access to the health system, education, distributive income, cigarette and alcohol consumption, and SDI.^23^ CoDCorrect is an algorithm to adjust CODEm's data to fit the specific mortality for each group, sex, year, and location from all GBD causes.^24^

According to the International Classification of Diseases (ICD), diseases are classified by a system of diagnostic codes used widely as a reference tool in cancer registries. In this study, LC only includes ICD‐10 codes C30 and C34.

### 2.3 Study variables

This study estimated the incidence, mortality, prevalence, YLLs, YLDs, and DALYs of LC and their trend in Iran. YLDs were calculated as the prevalence of disability multiplied by corresponding disability weight. YLLs due to LC were calculated from the number of age‐specific cancer deaths multiplied by the reference normative global life expectancy. LC DALYs were calculated by summing YLDs and YLLs.^25^ In the current study, we described mortality, prevalence, YLLs, YLDs, and DALYs of LC by age, sex, year, and location, and SDI. SDI values range from 0 to 100 (low SDI to high SDI). Quintiles are used for identifying locations with low, low‐middle, middle, middle‐high, and high SDI. The GBD classifies risks into four levels. Risks at level 1 include environmental, occupational, and metabolic risks. At Level 2, there are six risk categories, including tobacco, air pollution, occupational risks, High fasting blood plasma glucose, dietary risks, and other environmental risks. Level 3 risks include more specific risks such as smoking, second‐hand smoking, residential radon, occupational carcinogens, air pollution, diet low in fruits, high fasting blood plasma glucose. Level 4 risks are the most detailed classification which includes 18 risk categories.^26^ We examined deaths and DALYs attributable to GBD 2019 risk factors at Level 3. Risk factors in this paper include smoking, second‐hand smoking, residential radon, occupational carcinogens, air pollution, diet low in fruits, and high fasting blood plasma glucose. This study compared DALYs and death attributable to risk factors in 31 different provinces in Iran. All data were reported as all‐ages numbers and age‐standardized rates per 100,000 with 95% uncertainty interval (UI). For describing the disparities in 31 provinces, we calculated the Max/Min ratio in both 1990 and 2019 for all parameters (age‐standardized incidence, death, DALYs) for men and women and both sexes combined. If there was not a significant difference among them, we just reported the max and min in all parameters among all 31 provinces.

### 2.4 Statistical analysis

Calculations were performed for age groups of 15–49, 50–69, ≥ 70 years. Calculations were performed for 31 provinces of Iran with different SDI (higher values of SDI implying a higher level of development). For rate calculation, the total number of LC parameters (including incidences, mortality, and DALYs) was divided by the population over a specific period and expressed in 100,000 populations. Age‐standardized rates, including ASIR (age‐standardized incidence rate) and ASDR (age‐standardized death rate), were calculated using the GBD standard world population. To investigate the role of the population aging, population growth, and changes in age‐specific rates on incident cases of LC, decomposition analysis of the increase in absolute number of incidences of LC between 1990 and 2019, two scenarios were modeled: (1) Applying population age‐structure and age‐specific incidence rates of LC in 1990 into the population size of 2019, (2) and age‐specific LC incidence rates in 1990 alone into the 2019 age structure and population size. Differences between the estimated incidence of LC in 1990 and the first scenario were assigned to population growth. The disparity between the two scenarios was assigned to the changes in population age structure.^25^

## 3 RESULTS

**FIGURE 1:**
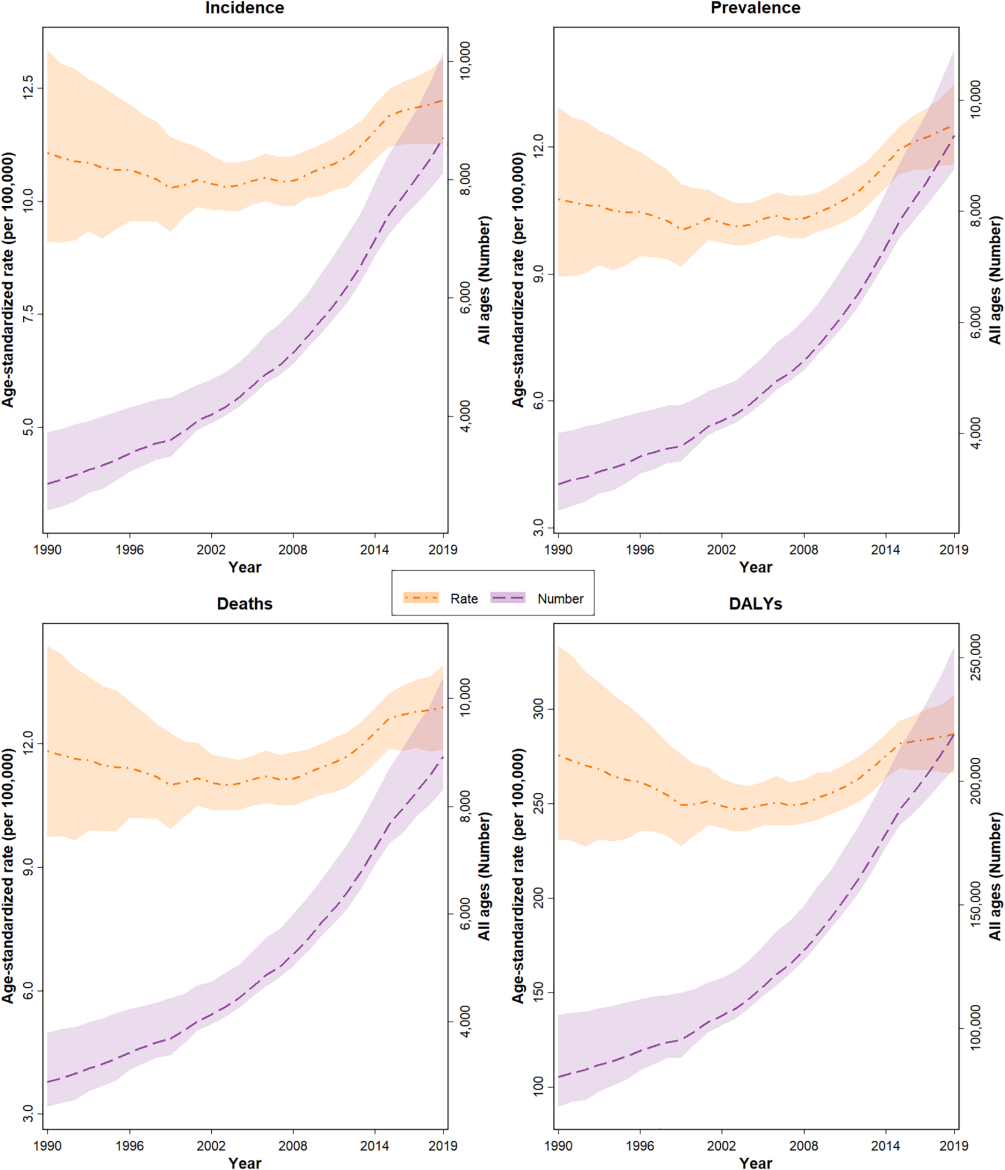
The trend of rate and number of the age‐standardized incidence, death, prevalence, and DALYs due to LC per 100,000 population during 1990–2019 in Iran with a 95% uncertainty interval.

**FIGURE 2:**
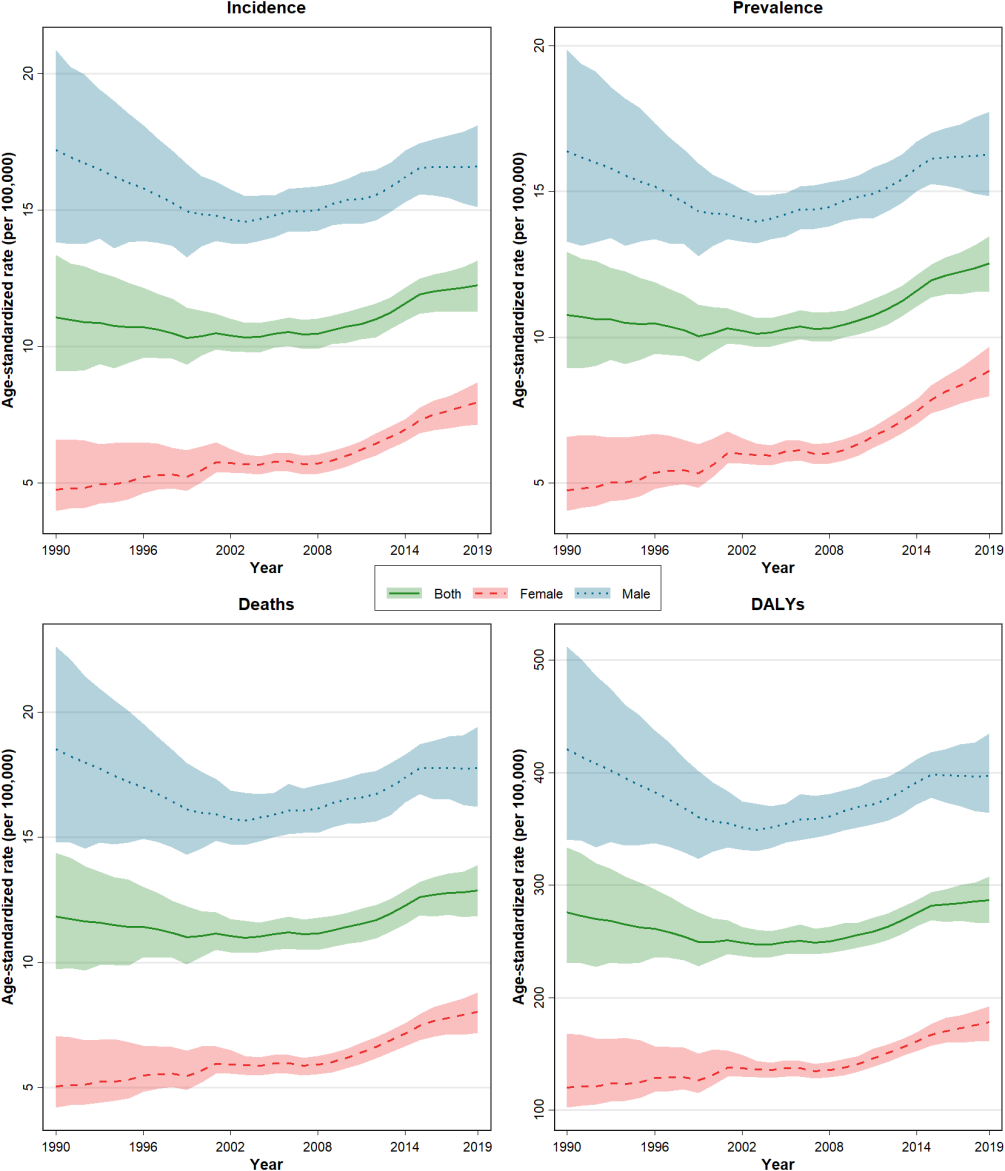
The trend of rate and number of the age‐standardized incidence, death, prevalence, and DALYs due to LC per 100,000 population by sex during 1990 and 2019 in Iran.

The LC mortality number in Iran showed an increase from 2889 deaths to 8923 during the years 1990 to 2019. In men, the death number increased from 2304 in 1990 to 6155 in 2019. (Table 1) Iran presented an unremarkable increase in the ASDR due to LC, from 11.8 deaths rate per 100,000 (95% UI: 9.7–14.4) in 1990 to 12.9 (11.9–13.9) in 2019. (Figure 1 and Table 1) The ASDR among men was 18.5 deaths per 100,000 inhabitants (14.8–22.6) in 1990 and 17.8 (16.2–19.4) in 2019. In contrast, women exhibited an increase, from 5.0 deaths per 100,000 inhabitants (4.2–7.0) in 1990 to 8.0 (7.2–8.8) in 2019—an increase of 63%. The ASDR of LC among women had an increasing trend, leading to a considerable reduction in the M/F ratio of ASDR over 30 years from 3.7 in 1990 to 2.2 in 2019, thus narrowing the ASDR gap between the two genders (Figure 2 and Table 1).

**TABLE 1:** The changes of rate and number of deaths, DALYs, and incidence due to LC in both sexes and ages and age‐standardized rates in Iran with a 95% uncertainty interval in 1990 and 2019

		Year								
		1990			2019			% Change (1990 to 2019)		
Measure	Metric	Both	Female	Male	Both	Female	Male	Both	Female	Male
Incidence	All ages number	2865 (2393–3441)	590 (506–817)	2275 (1844–2760)	8705 (8040–9366)	2827 (2548–3081)	5878 (5357–6411)	203.8 (139.4–284.7)	379.2 (217.3–487.5)	158.3 (99.4–242.8)
	Age‐standardized rate (per 100,000)	11.1 (9.1–13.4)	4.7 (4–6.6)	17.2 (13.8–20.9)	12.2 (11.3–13.2)	7.9 (7.1–8.7)	16.6 (15.1–18.1)	10.6 (−13.1–41.9)	67.4 (10.9–108.5)	−3.5 (−25.2–28)
Prevalence	All ages number	3080 (2586–3688)	670 (578–926)	2410 (1960–2920)	9366 (8689–10,053)	3337 (3012–3652)	6029 (5484–6596)	204.1 (140.3–280.5)	398.2 (228.2–503.4)	150.1 (93.1–229.7)
	Age‐standardized rate (per 100,000)	10.8 (8.9–12.9)	4.8 (4.1–6.6)	16.4 (13.3–19.9)	12.5 (11.6–13.5)	8.8 (8–9.7)	16.3 (14.8–17.7)	16.4 (−8.2–47.9)	85.8 (21.5–129.2)	−0.7 (−23.3–31.5)
Deaths	All ages number	2889 (2412–3501)	584 (495–816)	2304 (1857–2815)	8923 (8247–9595)	2768 (2488–3018)	6155 (5636–6725)	208.9 (141.8–296.5)	373.7 (208.4–484.7)	167.1 (104.4–253.7)
	Age‐standardized rate (per 100,000)	11.8 (9.7–14.4)	5 (4.2–7.1)	18.5 (14.8–22.6)	12.9 (11.9–13.9)	8 (7.2–8.8)	17.8 (16.2–19.4)	8.9 (−14.4–40.8)	59 (3.7–99.2)	−4 (−26.2–27.9)
DALYs	All ages number	80,596 (67,976–97,081)	17,355 (14,966–24,193)	63,240 (51,519–77,197)	218,990 (203,461–234,523)	68,653 (62,555–74,042)	150,337 (137,471–164,431)	171.7 (113.4–241.5)	295.6 (159.8–374.1)	137.7 (81.1–212.8)
	Age‐standardized rate (per 100,000)	275.8 (230.9–333.4)	120.1 (102.5–167.9)	420.7 (340.3–512)	286.8 (266.1–307.6)	178.2 (161.4–192.4)	396.9 (364.1–434.8)	4 (−18.2–32.5)	48.3 (−3.3–80.9)	−5.7 (−27.7–24.6)
YLLs	All ages number	79,920 (67,388–96,320)	17,208 (14,836–23,990)	62,712 (51,072–76,576)	216,967 (201,652–232,284)	67,974 (61,888–73,250)	148,993 (136,395–162,940)	171.5 (113.1–241.1)	295 (159.4–373.6)	137.6 (81–212.8)
	Age‐standardized rate (per 100,000)	273.3 (228.8–330.6)	119 (101.6–166.4)	416.9 (337.5–508.1)	284 (263.3–304.5)	176.3 (159.6–190.5)	393.2 (360.8–430.7)	3.9 (−18.2–32.4)	48.1 (−3.4–80.8)	−5.7 (−27.8–24.6)
YLDs	All ages number	675 (459–919)	147 (98–212)	529 (354–724)	2023 (1445–2617)	679 (482–882)	1344 (957–1756)	199.5 (135.5–273.6)	362.7 (205.3–468.6)	154.3 (94–238.1)
	Age‐standardized rate (per 100,000)	2.5 (1.7–3.4)	1.1 (0.7–1.6)	3.8 (2.6–5.3)	2.8 (2–3.6)	1.9 (1.3–2.4)	3.7 (2.7–4.8)	11 (−13–40.5)	65 (9–105.9)	−2.7 (−25.5–28.6)

*Note*: Data in parentheses are 95% Uncertainty Intervals (95% UIs).

Abbreviations: DALYs, disability‐adjusted life years; YLLs, years of life lost; YLDs, years lived with disability.

**FIGURE 3:**
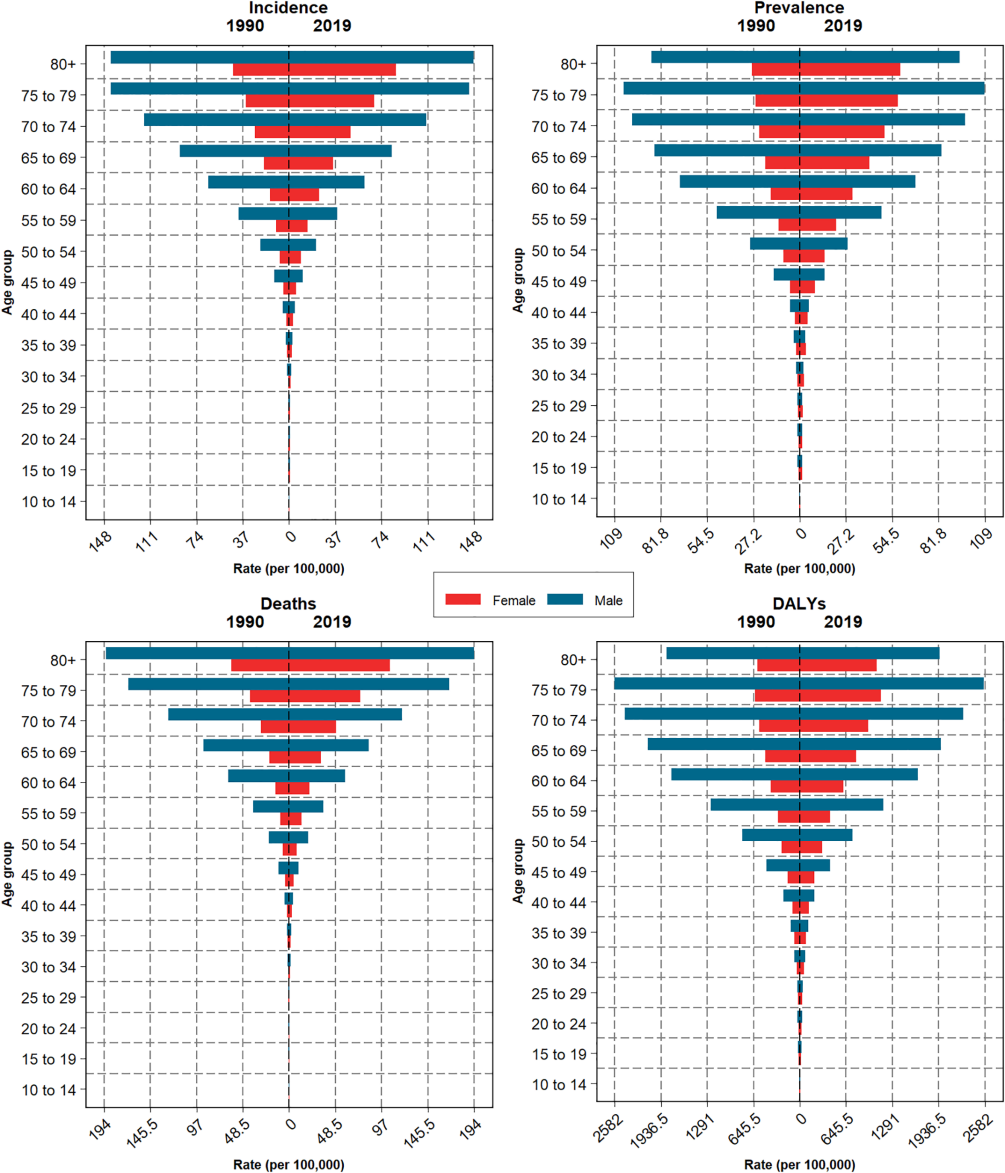
The DALYs, death, prevalence, and incidence rate of LC by age and sex with a 95% uncertainty interval between 1990–2019 in Iran.

The number of new cases of LC of all ages during the years 1990 and 2019 significantly increased from about 2865 (2393–3441) to 8705 (8040–9366) in both sexes (Table 1). During the same period, the incidence numbers of LC steadily increased in women and men of all ages from 590 (506–817) to 2827 (2548–3081) and from 2275 (1844–2760) to 5878 (5357–6411), respectively, while this increase was remarkable in women (Table 1). During the years 1990 and 2019, the ASIR of LC increased from 11.1 (9.1–13.3) per 100,000 to 12.2 (11.3–13.2) (Figure 1 and Table 1). The greatest incidence rate of LC was in the age group older than 70, and the rest mostly occurred in the age group of 50–69; the incidence rate of LC in the age groups older than 70 years was significantly higher than the younger age groups. The incidence rate of LC (+70 years old) was 79.5 (63.2–96.8) per 100,000 population in 1990 and increased to 98.9 (88.1–107.4) in 2019 (Table 2). The incidence rate of LC (+70 years old) was 65.9 (57.1–73.2) and 132.2 (118.4–144.9) per 100,000 population in females and males, respectively, in 2019 (Figure 3 and Table 2). ASIR in women had increased from 4.7 per 100,000 (4.0–6.6.0) to 7.9 (7.1–8.7), but in men had declined from 17.2 new cases per 100,000 (13.8–20.9) to 16.6 (15.1–18.1) (Figure 2 and Table 1). The increasing trend of the incidence rate of LC among women led to a considerable decline in the M/F ratio of the incident rate over 30 years from 3.6 in 1990 to 2.1 in 2019, thus reducing the incidence gap between the two sexes (Table 1). A similar pattern of LC incidence was observed in subnational analysis, except Qom, Kurdistan, Kermanshah, and Markazi provinces, which had a declining trend (Table S1).

**TABLE 2:** The changes of rate and number of deaths, DALYs, and incidence due–LC in different age groups in both sexes between 1990–2019

			Year								
			1990			2019			% Change (1990–2019)		
Age	Measure	Metric	Both	Female	Male	Both	Female	Male	Both	Female	Male
10–14	Incidence	Number	10 (9–13)	5 (4–7)	5 (5–7)	8 (6–11)	5 (4–6)	3 (2–5)	−23.1 (−44.9–4.5)	−1.6 (−37.4–36.6)	−42.4 (−63.5 to −5.2)
		Rate (per 100,000)	0.1 (0.1–0.2)	0.1 (0.1–0.2)	0.1 (0.1–0.2)	0.1 (0.1–0.2)	0.2 (0.1–0.2)	0.1 (0.1–0.1)	−8.5 (−34.4–24.5)	17.6 (−25.1–63.3)	−31.6 (−56.7–12.5)
	Deaths	Number	8 (7–10)	4 (3–5)	4 (4–5)	6 (5–8)	3 (3–4)	2 (2–4)	−27.2 (−47.7 to −0.6)	−9.2 (−41.0–27.0)	−43.0 (−63.7 to −4.8)
		Rate (per 100,000)	0.1 (0.1–0.1)	0.1 (0.1–0.1)	0.1 (0.1–0.1)	0.1 (0.1–0.1)	0.1 (0.1–0.1)	0.1 (0.1–0.1)	−13.3 (−37.7–18.4)	8.5 (−29.5–51.8)	−32.4 (−56.9–13.0)
	DALYs	Number	626 (512–764)	293 (219–392)	333 (272–402)	456 (353–604)	266 (206–330)	189 (132–289)	−27.2 (−47.7–−0.6)	−9.2 (−41.0–27.0)	−43.1 (−63.7–−4.9)
		Rate (per 100,000)	8.3 (6.8–10.1)	7.9 (5.9–10.6)	8.6 (7.1–10.4)	7.2 (5.6–9.5)	8.6 (6.6–10.7)	5.8 (4.1–8.9)	−13.3 (−37.7–18.3)	8.5 (−29.5–51.7)	−32.4 (−57.0–12.8)
15–49	Incidence	Number	413 (361–486)	135 (116–187)	277 (232–334)	1147 (1054–1252)	455 (414–498)	691 (613–784)	177.9 (121.3–236.3)	236.6 (116.9–308.2)	149.3 (89.5–230.4)
		Rate (per 100,000)	1.6 (1.4–1.8)	1.0 (0.9–1.4)	2.1 (1.7–2.5)	2.4 (2.2–2.6)	2.0 (1.8–2.1)	2.9 (2.5–3.3)	55.4 (23.8–88.1)	89.1 (21.8–129.3)	38.8 (5.6–84.0)
	Deaths	Number	359 (313–422)	115 (99–161)	244 (204–295)	986 (903–1072)	370 (334–402)	616 (548–694)	174.8 (119.4–234.5)	221.7 (107–287.1)	152.8 (91.5–233.6)
		Rate (per 100,000)	1.4 (1.2–1.6)	0.9 (0.8–1.2)	1.8 (1.5–2.2)	2.1 (1.9–2.3)	1.6 (1.4–1.7)	2.6 (2.3–2.9)	53.7 (22.7–87.1)	80.7 (16.2–117.4)	40.8 (6.7–85.8)
	DALYs	Number	18,131 (15,837–21,254)	5945 (5117–8311)	12,187 (10,235–14,756)	47,927 (43,965–52,183)	18,341 (16,555–19,929)	29,586 (26,295–33,383)	164.3 (111.3–220.0)	208.5 (99.5–270.3)	142.8 (84.3–218.8)
		Rate (per 100,000)	68.6 (59.9–80.4)	45.6 (39.3–63.8)	90.9 (76.3–110.1)	101.4 (93.0–110.4)	79.0 (71.4–85.9)	122.9 (109.2–138.7)	47.8 (18.2–79.0)	73.3 (12.0–108.0)	35.2 (2.6–77.5)
50–69	Incidence	Number	1678 (1378–2029)	295 (247–407)	1383 (1104–1689)	4118 (3803–4436)	1218 (1102–1337)	2900 (2629–3182)	145.3 (91.6–214.7)	312.7 (170.3–417.8)	109.6 (60.5–183.1)
		Rate (per 100,000)	32.1 (26.4–38.8)	12.2 (10.2–16.8)	49.4 (39.4–60.3)	31.3 (28.9–33.7)	18.5 (16.7–20.3)	44.2 (40.1–48.5)	−2.5 (−23.8–25.1)	51.8 (−0.6–90.4)	−10.4 (−31.4–21.0)
	Deaths	Number	1637 (1336–1991)	283 (235–401)	1354 (1074–1663)	3895 (3615–4188)	1097 (1002–1191)	2798 (2548–3064)	138.0 (84.3–208.7)	287.9 (147.8–387.1)	106.7 (56.0–176.2)
		Rate (per 100,000)	31.3 (25.5–38.1)	11.7 (9.7–16.5)	48.3 (38.3–59.3)	29.6 (27.5–31.8)	16.6 (15.2–18.0)	42.7 (38.9–46.7)	−5.4 (−26.7–22.7)	42.6 (−8.9–79.1)	−11.6 (−33.3–18.1)
	DALYs	Number	47,458 (38,921–57,842)	8345 (6983–11,796)	39,113 (31,153–48,038)	113,902 (105,551–122,555)	31,966 (29,231–34,745)	81,937 (74,643–89,950)	140.0 (86.6–211.0)	283.1 (145.8–377.2)	109.5 (58.3–179.3)
		Rate (per 100,000)	907.5 (744.3–1106.1)	343.9 (287.8–486.2)	1395.5 (1111.5–1713.9)	865.9 (802.4–931.7)	484.5 (443–526.6)	1249.8 (1138.6–1372)	−4.6 (−25.8–23.6)	40.9 (−9.6–75.5)	−10.4 (−32.3–19.4)
70+	Incidence	Number	764 (607–930)	155 (123–216)	609 (475–745)	3432 (3058–3728)	1149 (996–1277)	2283 (2046–2504)	349.3 (249.4–494.4)	643.0 (398.0–876.5)	274.8 (191.3–408.8)
		Rate (per 100,000)	79.5 (63.2–96.8)	32.6 (26.0–45.5)	125.2 (97.7–153.0)	98.9 (88.1–107.4)	65.9 (57.1–73.2)	132.2 (118.4–144.9)	24.3 (−3.3–64.5)	102.0 (35.4–165.5)	5.6 (−18.0–43.3)
	Deaths	Number	885 (711–1081)	183 (147–252)	702 (551–863)	4036 (3611–4386)	1298 (1124–1447)	2738 (2450–3019)	355.9 (253.7–503.7)	609.9 (365.1–840.2)	289.8 (201.1–429.5)
		Rate (per 100,000)	92.2 (74.0–112.5)	38.6 (31.0–53.1)	144.4 (113.3–177.3)	116.3 (104.0–126.3)	74.4 (64.4–83.0)	158.5 (141.8–174.7)	26.2 (−2.1–67.1)	93.0 (26.5–155.6)	9.8 (−15.2–49.1)
	DALYs	Number	14,380 (11,544–17,586)	2772 (2222–3817)	11,608 (9090–14,258)	56,705 (51,498–61,706)	18,080 (15,840–20,110)	38,625 (34,983–42,423)	294.3 (208.7–424.3)	552.2 (328.5–773.1)	232.8 (157.5–352.7)
		Rate (per 100,000)	1496.7 (1201.5–1830.4)	584.7 (468.7–805)	2385.4 (1868.1–2930)	1633.3 (1483.3–1777.3)	1036.7 (908.3–1153.1)	2235.4 (2024.6–2455.2)	9.1 (−14.6–45.1)	77.3 (16.5–137.4)	−6.3 (−27.5–27.5)

*Note*: Data in parentheses are 95% Uncertainty Intervals (95% UIs).

Abbreviation: DALYs, disability‐adjusted life years.

The decomposition analysis showed an increase in the incident cases of LC in Iran that 24.6%, 135.2%, and 44% of the total 203.8% rise in incident cases between 1990 and 2019 were due to the increase in the age‐specific incidence rate, population aging, and population growth, respectively (Table S2).

There were approximately 9366 (8689–10,053) prevalent cases of LC in 2019 with an increasing trend of 204.1% (140.3–280.5), with an age‐standardized prevalence rate (ASPR) of 12.5 (11.6–13.5) per 100,000 population in 2019. The percent change in the ASPR was 16.4 (−8.2 to 47.9) at the national level from 1990 to 2019 (Table 1).

According to the GBD, Iran presented a significant increase in DALYs number in all ages and sexes about 2.5 times. In women presented about four times increase and in men about 2.5 times increase (Table 1). From 1990 to 2019, Iran presented an unremarkable increase in the age‐standardized DALYs rate due to LC, from 275.8 DALYs rate per 100,000 (230.9–333.4) in 1990 to 286.8 (266.1–307.6) in 2019, which 99% of this burden came from YLLs (Table 1). The DALYs rate among men declined, from 420.7 DALYs rate per 100,000 (340.3–512.0) in 1990 to 396.9 (364.0–434.8) in 2019. In contrast, women exhibited an increase, from DALYs rate 120.1 per 100,000 (102.5–167.9) in 1990 to 178.2 (161.4–192.4) in 2019 (Figure 2 and Table 1).

**FIGURE 4:**
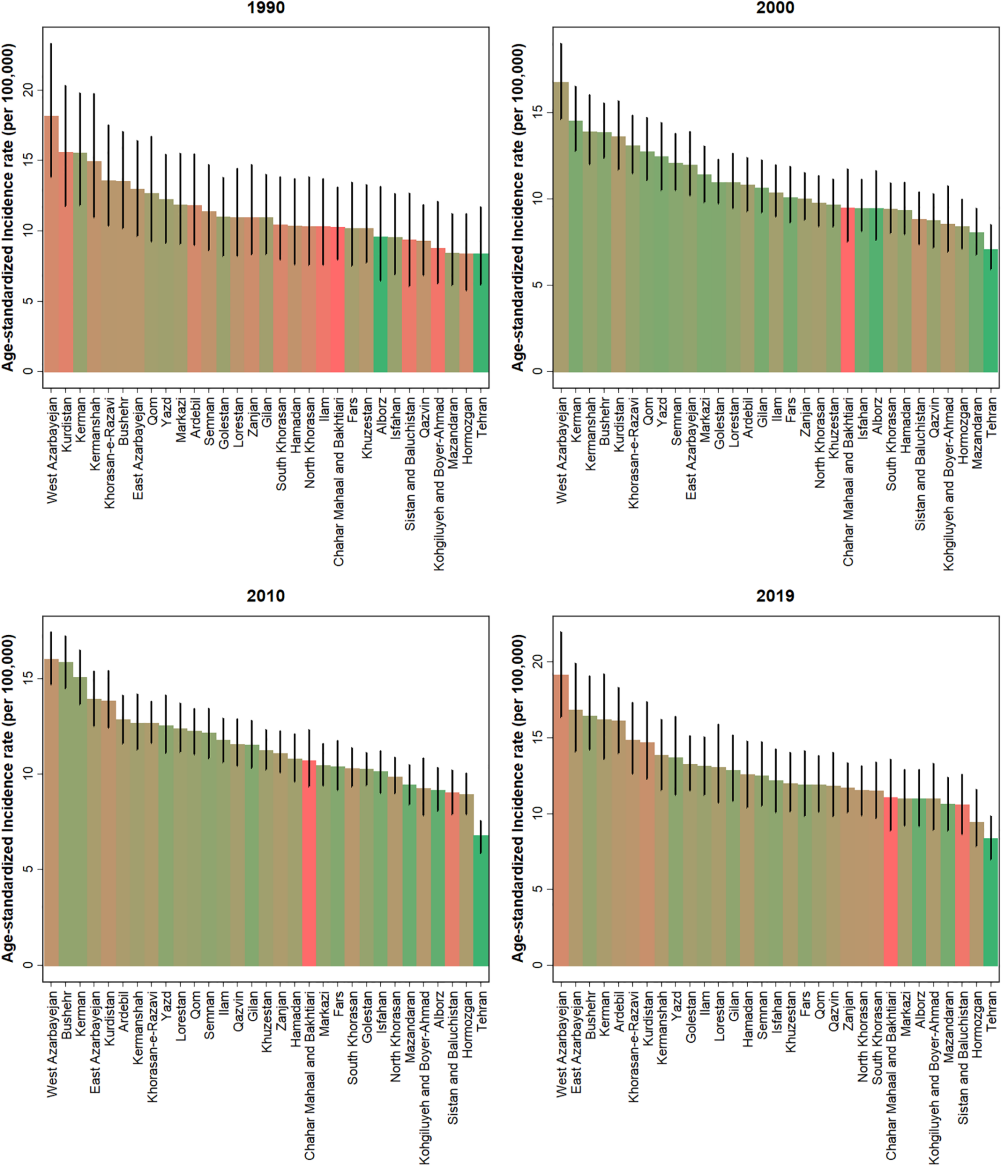
Age‐standardized incidence rate of LC in all 31 provinces of Iran by year with a 95% uncertainty interval.

**FIGURE 5:**
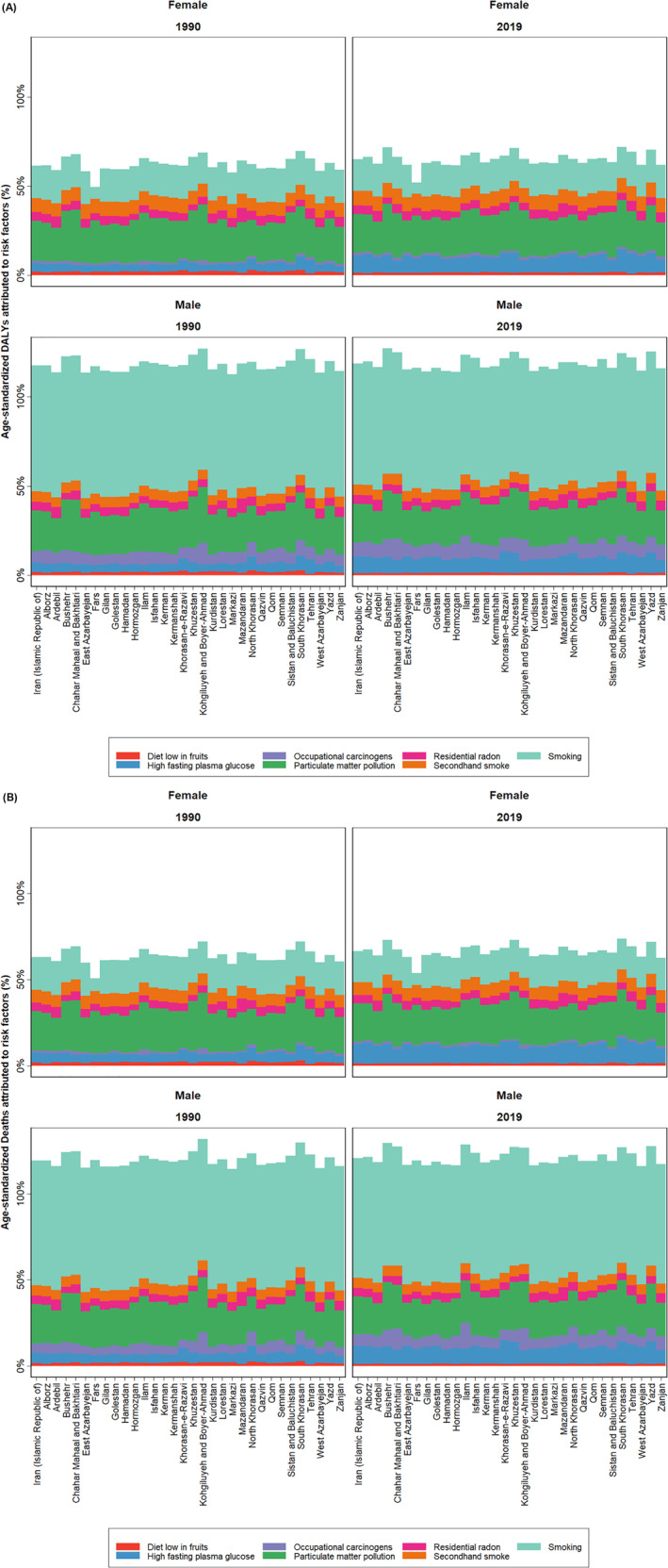
(A) Age‐standardized DALYs rate of LC due to risk factors by sex in all 31 provinces of Iran with a 95% uncertainty interval in 1990–2019. (B) Age‐standardized death rate of LC attributed to risk factors by sex in all 31 provinces of Iran with a 95% uncertainty interval in 1990–2019.

Data analysis at the sub‐national level indicated that there was not significant difference between Max/Min ratio among provinces in 1990 and 2019 in ASIR, ASDR, and age‐standardized DALYs rate. The current study showed the highest ASIR of LC in 2019 was observed in West Azarbayejan, at a rate of 19.1 (16.4–22.0) per 100,000 population of both sexes combined (Figure 4). The lowest ASIR was observed in Tehran, at 8.3 (7.0–10.0) per 100,000 population (Figure 4). The highest ASDR in 2019 was observed in West Azarbayejan 20.6 (17.7 to 24.0) per 100,000 population, and the lowest was observed in Tehran, 8.7 (7.3 to10.3) per 100,000 population (Figure S1 and Table S1). The age‐standardized DALYs rate in 2019 had the same pattern with 439.0 DALYs rate per 100,000 (377.4–507.1) in West Azarbayejan and 185.1 DALYs rate per 100,000(155.4–218.5) in Tehran (Figure 5A). In all decades, the incidence rate of LC, West Azarbayejan was in the top rank, and Tehran was in the bottom ranking (Figure 4). The mortality and DALYs rates of LC in 2019 were not changed significantly across the provinces with different SDI.

In 1990, the DALYs rate in one of the highest SDI (Kerman) was 389.3 DALYs per 100,000 (295.5–505.5), in contrast in the provinces within one of the lowest (Kohgiluye and Boyer‐Ahmad) SDI, it was 222.1 per 100,000 (155.8–313.7). In 2019, the average DALYs in the highest and lowest SDI were approximately equal. (Alborz's DALYs rate as higher SDI is 246.5 per 100,000 (208.9–290.9) and Chahar Mahaal and Bakhtiari is 254.3 per 100,000 (202.6–315.1) (Figure S2). In all provinces of Iran, new cases of LC among men are more than in women (Table S1). The LC mortality, DALYs, and death rate in all provinces revealed a stable trend in both genders, the maximum to minimum ratio (Table S1).

Regarding the mortality rate by risk factor, GBD showed that smoking with 53.46% of total deaths (51.0%–55.9%) remained the leading risk factor (Table 3) with 7.2 per 100,000 deaths (5.8–8.9) by LC attributable to smoking in 1990 and 6.9 deaths per 100,000 (6.3–7.6) in 2019. In men, smoking was the first leading risk factor from 1990 to 2019, with 13.4 deaths and 12.4 per 100,000 of LC attributable to smoking in 1990 and 2019, respectively. Conversely, in women, smoking was the second risk factor for LC and showed an increase, caused 1.0 death per 100,000 in 1990 and 1.4 deaths per 100,000 in 2019. So, in men, smoking was a more significant mortality risk factor than women (Figure 5B).

**TABLE 3:** Age‐standardized death rate and percent of LC due to risk factors by sex with a 95% uncertainty interval in 1990–2019

		Year								
		1990			2019			% Change (1990–2019)		
Risk factor	Metric	Both	Female	Male	Both	Female	Male	Both	Female	Male
Diet low in fruits	Rate	0.2 (0.0–0.3)	0.1 (0.0–0.2)	0.3 (0.1–0.5)	0.2 (0.0–0.2)	0.1 (0.0–0.2)	0.2 (0.1–0.3)	−23.4 (−44.4–6.6)	11.4 (−29.6–67.5)	−34.3 (−56.6–0.0)
	Percent	1.7 (0.5–2.5)	1.9 (0.5–2.8)	1.6 (0.4–2.5)	1.2 (0.4–1.9)	1.3 (0.4–2.2)	1.1 (0.3–1.8)	−29.7 (−44.2 to −8)	−29.9 (−48.4 to −6.5)	−31.7 (−49.2 to −5.9)
High fasting plasma glucose	Rate	0.7 (0.1–1.7)	0.3 (0.1–0.7)	1.1 (0.2–2.8)	1.4 (0.3–3.0)	0.9 (0.2–2.1)	1.8 (0.3–4.2)	94.6 (51–162.4)	214.4 (114–320.8)	63.9 (26.7–126)
	Percent	6.0 (1.3–13.6)	5.7 (1.1–13.7)	6.1 (1.0–14.6)	10.7 (2.5–22.8)	11.3 (2.3–25.2)	10.4 (1.8–23.5)	78.8 (62.8–117.4)	98.2 (83.2–122.2)	70.8 (60.2–87.6)
Occupational carcinogens	Rate	0.5 (0.3–0.8)	0.1 (0.0–0.1)	1.0 (0.6–1.4)	0.6 (0.4–0.9)	0.1 (0.1–0.1)	1.2 (0.8–1.6)	21.0 (−10.8–62.6)	57.5 (3.1–127.1)	24.9 (−9.4–69.0)
	Percent	4.5 (3.0–6.2)	1.3 (0.9–1.7)	5.1 (3.4–7.2)	5.0 (3.5–6.7)	1.3 (0.9–1.7)	6.7 (4.7–9.0)	11.0 (−5.3–35.3)	−1.0 (−17.4–20.3)	29.9 (8.2–55.9)
Particulate matter pollution	Rate	2.7 (1.9–3.7)	1.2 (0.8–1.7)	4.3 (3.0–5.9)	2.8 (2.1–3.6)	1.8 (1.3–2.3)	3.9 (3.0–5.0)	4.8 (−18.1–36.2)	53.7 (0.9–92.9)	−7.7 (−29.0–24.3)
	Percent	23.0 (17.4–28.6)	22.9 (17.3–28.6)	23.0 (17.4–28.6)	22.1 (16.4–27.7)	22.2 (16.5–27.6)	22.1 (16.4–27.6)	−3.7 (−7.4 to −1.4)	−3.3 (−7.5 to −0.6)	−3.8 (−7.4 to −1.4)
Residential radon	Rate	0.6 (0.1–1.2)	0.2 (0.0–0.5)	0.9 (0.2–1.9)	0.6 (0.1–1.3)	0.4 (0.1–0.8)	0.9 (0.2–1.8)	9.7 (−14.1–41.9)	60.4 (5.0–102.6)	−3.4 (−26.2–29.6)
	Percent	4.9 (1.0–9.9)	4.9 (1.0–10.0)	4.9 (1.0–9.8)	4.9 (1.0–9.9)	4.9 (1.0–10.1)	4.9 (1.0–9.9)	0.8 (−4.6–6.3)	0.6 (−4.8–7.1)	0.8 (−5.6–7.6)
Secondhand smoke	Rate	0.7 (0.4–1.1)	0.4 (0.2–0.6)	1.1 (0.6–1.7)	0.8 (0.5–1.2)	0.6 (0.4–0.9)	1.0 (0.6–1.6)	11.6 (−13.9–45.9)	63.2 (9.3–110.1)	−5.2 (−29.4–30.9)
	Percent	6.2 (3.7–9.1)	7.4 (4.4–10.8)	5.9 (3.5–8.8)	6.4 (3.8–9.3)	7.6 (4.5–11.1)	5.9 (3.4–8.8)	2.6 (−8.0–14.5)	2.7 (−7.2–13.1)	−1.1 (−14.7–14.8)
Smoking	Rate	7.2 (5.8–8.9)	1.0 (0.7–1.4)	13.4 (10.7–16.5)	6.9 (6.3–7.6)	1.4 (1.2–1.7)	12.4 (11.2–13.6)	−4.8 (−26.6–26.7)	50.0 (−2.5–118.1)	−8.0 (−29.5–23.5)
	Percent	61.1 (57–64.6)	19.0 (15.1–23.6)	72.5 (70.1–75)	53.5 (51.0–55.9)	18.0 (15.1–21.3)	69.5 (67–71.9)	−12.5 (−18.1–−4.8)	−5.7 (−26.0–22.2)	−4.1 (−7.4 to −0.6)

*Note*: Data in parentheses are 95% Uncertainty Intervals (95% UIs).

The ASDR from LC due to second‐hand smoking was 0.7 per 100,000 (0.4–1.1) in 1990 and 0.8 per 100,000 (0.5–1.2) in 2019, (Figure S3) in women, it had approximately 65% increase from 0.4 deaths (0.2–0.6) per 100,000 to 0.6 deaths per 100,000 (0.4–0.9) during 1990–2019, but in men did not have significant change (Figure 5B).

The ASDR from LC attributable to particulate matter pollution in both sexes was 2.7 per 100,000 (1.9–3.7) in 1990 and 2.8 per 100,000 (2.1–3.6) in 2019 but had a decline from 1990 to 2004 (2.5 death per 100,000) then increased again. Bushehr had the highest death rate from LC attributable to particulate matter pollution between different provinces in 30 years. After Bushehr, Kerman and West Azarbayejan had the highest rate (Figure S3).

ASDR and age‐standardized DALYs rate from LC attributable to particulate matter pollution among women were highest from 1990 to 2019, 1.8 per 100,000 (1.3–2.3) and 38.8 per 100,000 (28.5–49.0) in 2019, respectively (Table 3). During 30 years had a significant increase in both parameters (Table 3 and Table S3).

The death rate from LC attributable to high fasting blood plasma glucose showed a significant increase of about 94.6% from 0.7 per 100,000 (0.1–1.7) in 1990 to 1.4 per 100,000 (0.3–2.9) in 2019. In all provinces of Iran, smoking was the main risk factor for DALYs and death due to LC in both sexes. (Figure 5A,B and Table 3; Figure S3).

ASDR and age‐standardized DALYs rate from LC attributable to occupational carcinogen during all three decades had not any significant changes in men, women, and both sexes combined. ASDR and age‐standardized DALYs rate from LC attributable to occupational carcinogen were higher in men compared to women. ASDR were 1.2 per 100,000 (0.8–1.6) in men and 0.1 per 100,000 (0.1–0.1). Age‐standardized DALYs rate were 30.9 per 100,000 (21.2–42.2) in men and 2.6 per 100,000 (1.7–3.5) in 2019. There was not a significant change during 1990–2019 (Table 3; Table S3). Also, there was not significant difference between men and women in ASDR and age‐standardized DALYs rate from LC attributable to residential radon during 1990–2019 (Table 3; Table S3).

Among LC attributed risk factors, diet low in fruits had the lowest burden and death rate in men and women and both sexes combined. ASDR was 0.2 per 100,000 (0.0–0.2) and age‐standardized DALYs rate was 3.5 per 100,000 (1.0–5.5) in both sexes combined. Also, during 30 years there was not any significant change in death or DALYs rate (Table 3; Table S3).

## 4 DISCUSSION

The current study is a population‐based epidemiological study performed on LC burden based on the GBD 2019 data in Iran. This study revealed an update of mortality and DALYs rate for disease burden due to LC in Iran by age, sex, risk factor, and SDI. The study showed that the highest incidence and DALYs rate of LC were in the age group >70. There were considerable inequalities between provinces with the highest and lowest rates of incidence, mortality, and disability. Age‐standardized incidence and DALYs and death rate in females were lower than males but had a remarkable increase. The LC incidence, mortality, and DALYs rate had an unremarkable increase in the last decades. Smoking was the first leading risk factor among the overall population, men, and all provinces of Iran.

The LC incidence, mortality, and DALYs rate had increased in the last decades. In contrast, in 2016, Rajai et al. showed a decline in lung cancer mortality rate.^4^ The growing trend of these parameters in LC in the entire population of Iran demands a rapid application of preventive approaches and policies. Unlike the approval of the Framework Convention on Tobacco Control (FCTC), a comprehensive strategy for tobacco control, based on the MPOWER measures, but this legislation has not been achieved yet.^10^ The MPOWER (Monitoring tobacco use, Protect people consumption, Offer help to quit smoking, Warn, Enforce bans, Raise taxes) package consists of six evidence‐based and proven policies to encounter the global prevalence of tobacco. These approaches have been shown to rebate smoking usage conclusively.^27^ So, this increasing trend in LC incidence could result from not achieving this legislation, which necessitates the critical cancer control policies. On the other hand, chemotherapy increased survival and standards of living in patients with LC.^28^ In addition, advancements in LC screening technology, diagnosis, and treatment approaches have led to improved survival,^29^ and these improved survival periods could result in the growing trend of LC DALYs. This lack of achievement of the FCTC goals, necessitates appropriate cultural policies that target smoking control measures to reduce the burden of LC in Iran. For example, one of the types of smoking methods, water pipes (hookah) are commonly used in Iran, it emphasizes the necessity of cultural sensitivity when it comes to improving smoking control strategies in these settings.^30^

We found an increase in age‐standardized DALYs rate during 30 years which is 99% of that came from YLLs. This indicates LC causes more deaths than disabilities which imposes need to early diagnosis and screening, curative therapeutic options, and sufficient health systems.

Age‐standardized incidence and DALYs and death rate in females were lower than males but had a remarkable increase. The increasing trend of the incidence rate of LC among women led to a considerable decline in the M/F ratio of the incident rate over 30 years from 1990 to 2019. This trend is similar to the result on the global level.^31^ Women are more careful about their healthcare; therefore, their diagnosis and treatment for LC occur at the onset of the first signs^32^; in addition, smoking in women has a stigma in Iran hence smoking is lower among women,^33^ which could be the reasons for lower deaths and incidence and DALYs rates of LC in women than men. According to WHO 2010, although smoking prevalence rates were lower among women than men, they were at increased risk of tobacco consumption in many low‐ and‐ middle‐income countries.^34^ As a middle‐income country, Iran had an increasing pattern in death and DALYs rates in women and reducing the incidence gap between genders could be attributed to the upswing prevalence of smoking among women in Iran, which could increase the incidence of LC among women.

Another finding in this study was the clear geographical difference and regional varieties in DALYs rate due to LC; West Azarbayejan shows a higher DALYs rate than the rest. In 2019, West Azarbayejan left the highest DALYs rate, and Tehran had the lowest DALYs rate due to LC. In comparison, Rajai et al. reported that the highest and lowest ASDR in 2016 were in Alborz and Sistan and Baluchistan, respectively.^4^ LC is a multifactorial disease that makes different rates and disparities in the different provinces of Iran. Cancer is caused by a combination of genetic and environmental factors that effectively make provincial variations.^35^ The western provinces of Iran have higher incidence rates of LC; this might result from a higher level of dust and air pollutants in these provinces.^5^

This study showed males and older (>70) carried the majority burden of LC, this might be due to the higher prevalence of smoking in older men than women,^13^ and starting smoking at a younger age in men compared to women.^36^ Also, in Iran, consistent with a previous study, men start smoking at an earlier age, smoke more cigarettes than women, and increase this habit by increasing age.^33^ By age group, LC mortality, incidence, DALYs rates are higher at advanced ages (>70 years) that can be related to the population aging (65 and over) and growth worldwide.^37^

GBD showed that smoking remained the leading risk factor in the overall population and men regarding the mortality rate attributed to risk factors. Conversely, in women, smoking was the second leading risk factor for LC. So, in men, smoking was a more significant mortality risk factor than women. Tobacco smoking is the main leading risk factor of LC, and cigarettes are the main tobacco product using in western countries.^8^ Smoking termination reduces the incidence of LC; however, even lessening the number of cigarettes smoked daily has a benefit.^38^ Tobacco control policies in Iran have not been employed successfully.^39^ The rate of using tobacco products is higher among men than women.^39^ The prevalence of smoking is about 12.5%, 24.4%, and 3.8% in all populations, males, and females in Iran, respectively.^13^, ^40^ The prevalence of cigarette smoking among Iranian women may be underestimated due to the culture that shows smoking as an unfavorable and stigma act for women and leads to women hide their smoking.^41^ The present study showed that the death rate from LC attributed to high fasting blood plasma glucose showed a significant increase of about 94.3%. Fasting plasma glucose levels might have a consequence on the prognosis of LC especially squamous cell carcinoma.^42^ Metabolic syndrome has an impact on increasing the risk of LC in men. Also, several components of metabolic syndrome are associate with the higher risk of LC.^43^ In Iran, one‐third of adults have metabolic syndrome, and its rate is higher in women than men.^44^ Khosravi‐Boroujeni et al. reported a 6.9% increase in the prevalence of metabolic syndrome in Iran over 12 years.^45^ Therefore, this case could be why the death rate from LC attributed to high fasting blood plasma glucose has been increased.

According to our study, the death rate from LC due to second‐hand smoking (SHS) increased insignificantly during 1990–2019, and in women had a 63% increase. A history of SHS in adults in never‐smokers had a possibility of a later diagnosis of LC and also death from LC.^46^ Globally in adults, the level of SHS exposure was higher among females than in males.^47^ In the USA, levels of SHS exposure among nonsmokers have reduced, which may be due to increased knowledge of social concerns about the health effects of SHS and strategies to control smoking in public places, which decreases cigarette smoking rates.^48^ Obviously, Smoking and SHS exposure have a direct correlation,^47^ but in Iran, MPOWER has not achieved goals yet; this increase in our study can be because of this. So, it can increase secondhand smoke. The higher prevalence of smoking in men than women in Middle Eastern countries such as Iran puts women at risk for SHS, although tobacco smoking in women is low.^49^ Furthermore, smoking behaviors among Iranian adults are increasing.^50^

ASDR and age‐standardized DALYs rate from LC attributable to occupational carcinogen were higher in men compared to women. A previous study showed about 90% of women are in service sectors but just about 21% of men are in this sector. Men were most likely to work in agriculture and manufacturing which result in more exposure to occupational carcinogens.^51^

The present study showed a decline from 1990 to 2004, then increased again in the death rate from LC attributable to particulate matter pollution. Air pollution is on the rise in developing countries like Iran due to industrial activities and rising emissions such as unsuitable vehicles, and outdoor air pollution is thought to increase the risk of LC.^52^, ^53^ Another leading cause of air pollution is benzene, commonly added to gasoline.^54^ In early 2014, the government of Iran, because of sanctions, confirmed substandard gasoline formulation, which causes the production of increased levels of benzene.^55^ Additional efforts are needed to control and reduce air pollution.^56^ In this present study, Bushehr, Kerman, and West Azarbayejan had the highest death rate from LC attributable to particulate matter pollution between different provinces in 30 years. Shamsipour et al. reported that the level of concentration of particulate matter and its total death was higher in the western provinces of Iran than elsewhere.^14^

### 4.1 Strengths and limitations

The strength of our study is that we presented an updated burden of LC and its risk factors at the national and subnational scales. Since we depended on the GBD Study, specific issues must be considered. Although the GBD study generally provides good quality data on the burden of diseases, it has many limitations. One of the primary sources of data for public health is the death certificate, but the probability of variation in death rate and identification of the leading cause of death in this method is high. GBD 2019 follows the ICD principles to develop the comparability of health information coded and assigned a cause for each disease.^57^ The availability of raw data is one of the main limitations of the GBD study. If data are not available, the results will be based on the modeling predictability. The development of data modeling can improve the accuracy of the results, but more data is needed for a fundamental upgrade.^24^ Another limitation in GBD 2019 is lacking data related to other risk factors attributed to LC, such as SO2, which is one of the major occupational risks. Exposure to occupational carcinogens is associated with types of occupation. Still, GBD estimation has limitation and has just one pattern, and do not estimate based on types of occupational variation.

## 5 CONCLUSIONS

In summary, the present study showed that LC mortality and DALYs rates in Iran have increased during the years 1990 and 2019 and showed the provincial disparities. This burden of LC is concentrated in the advanced age groups. These results emphasize the urgent implementation of public health policies to considerably reduce the exposure to the risk factors and control LC.

## AUTHOR CONTRIBUTIONS

Zahra Shokri Varniab: conceptualization, formal analysis, methodology, investigation, writing—Original Draft. Yeganeh Sharifnejad Tehrani: formal analysis, data curation, visualization, software. Ashkan Pourabhari Langroudi: conceptualization, investigation, writing—Original Draft, writing – review & editing. Sina Azadnajafabad: validation, data curation, writing – review & editing. Negar Rezaei: validation, investigation, data curation, project administration, writing – review & editing. Mohammad‐Mahdi Rashidi: formal analysis, investigation, data curation, visualization. Zahra Esfahani: formal analysis, investigation, data curation. Mohammad‐Reza Malekpour: conceptualization, data curation. Erfan Ghasemi: conceptualization, validation, data curation. Azin Ghamari: validation, investigation. Arezou Dilmaghani‐Marand: investigation, data curation. Sahar Mohammadi Fateh: investigation, data curation. Alireza Namazi Shabestari: investigation, visualization. Bagher Larijani: conceptualization, methodology, writing – review & editing, investigation, validation. Farshad Farzadfar: conceptualization, funding acquisition, methodology, resources, writing – review & editing, supervision. All authors read and approved the final manuscript.

## FUNDING STATEMENT

Not applicable.

## CONFLICT OF INTEREST

The authors declare no competing interests.

## ETHICS APPROVAL AND CONSENT TO PARTICIPATE

The ethics committee of Endocrinology and Metabolism Research Institute at Tehran University of Medical Sciences approved the study with the reference number of IR.TUMS.EMRI.REC.1400.025.

## CONSENT FOR PUBLICATION

Not applicable.

## Supporting information


Figure S1

Figure S2

Figure S3

Table S1

Table S2

Table S3

## Data Availability

Data sharing is not applicable to this article as no new data were created or analyzed in this study.
