# Supplementary figures and images for: Estimates of incidence, prevalence, mortality, and disability‐adjusted life years of lung cancer in Iran, 1990–2019: A systematic analysis from the global burden of disease study 2019
Source: Cancer Med. 2022 Jun 13;11(23):4624–40. doi: 10.1002/cam4.4792 (PMC9741968; doi:10.1002/cam4.4792)

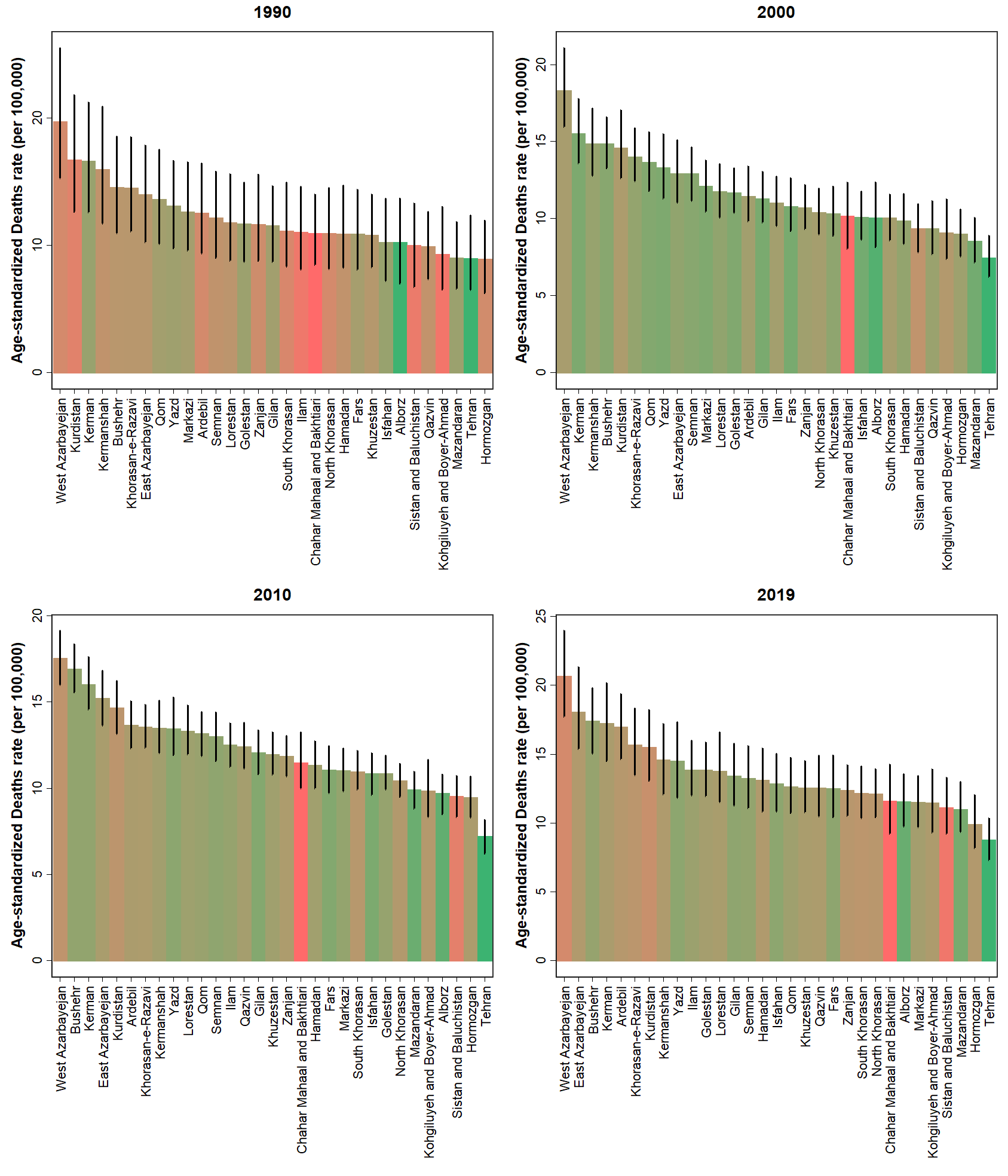

Supplement: Supplementary file 1 — Figure S1 [file CAM4-11-4624-s003.tiff]

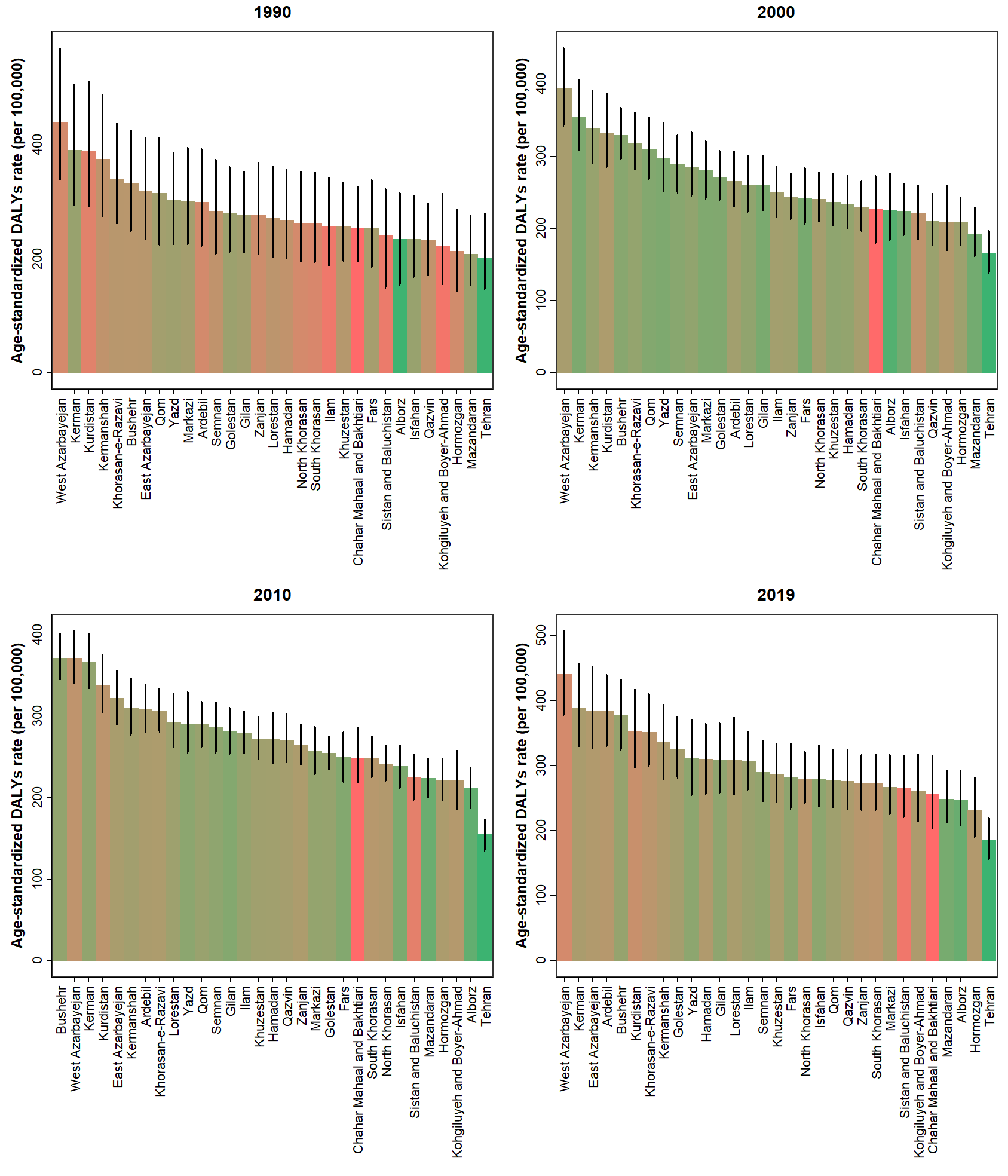

Supplement: Supplementary file 2 — Figure S2 [file CAM4-11-4624-s002.tiff]

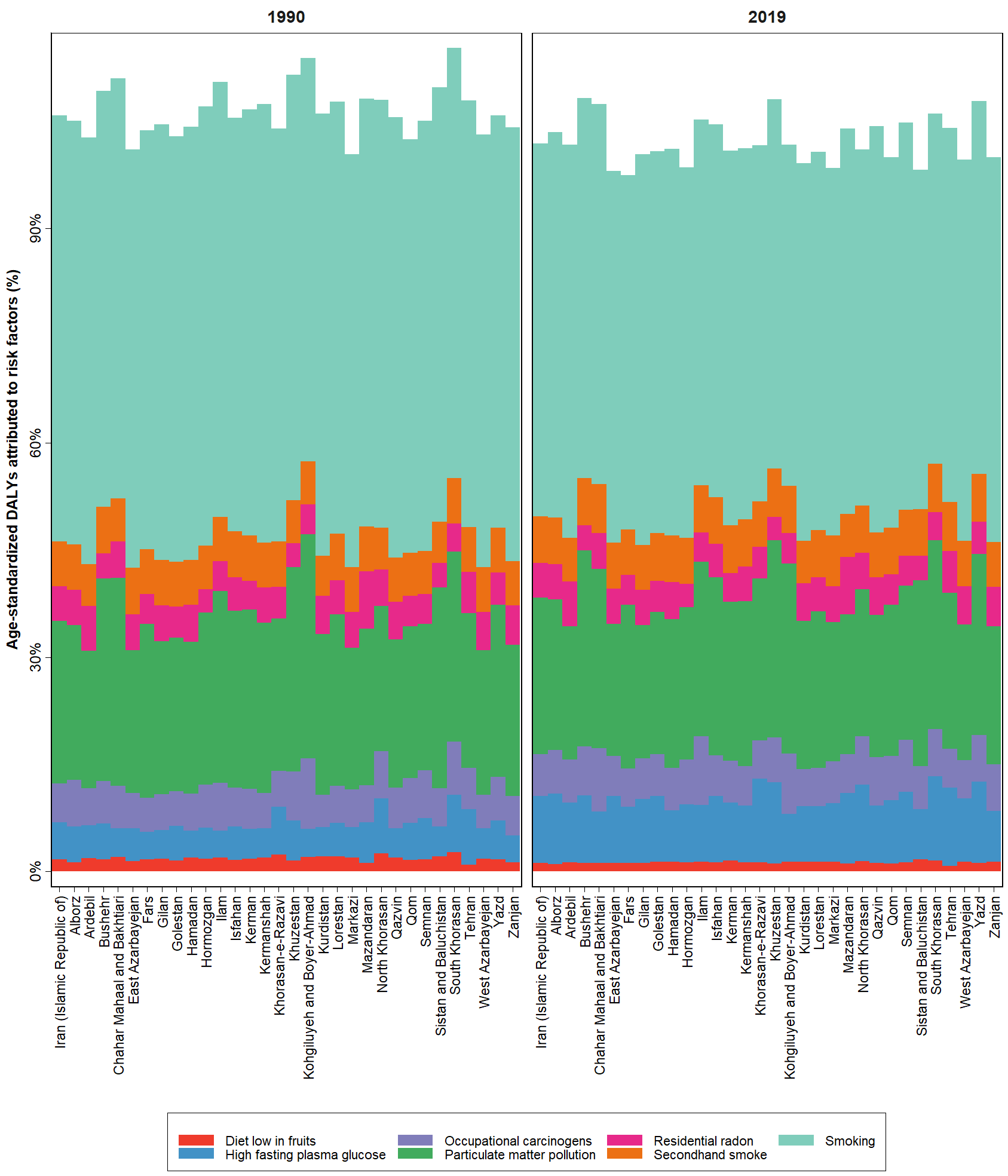

Supplement: Supplementary file 3 — Figure S3 [file CAM4-11-4624-s006.tiff]
