# Supplementary material for: Estimates of incidence, prevalence, mortality, and disability‐adjusted life years of lung cancer in Iran, 1990–2019: A systematic analysis from the global burden of disease study 2019
Source: Cancer Med. 2022 Jun 13;11(23):4624–40. doi: 10.1002/cam4.4792 (PMC9741968; doi:10.1002/cam4.4792)
Supplement: Supplementary file 4 — Table S1 [file CAM4-11-4624-s001.pdf]

Province	Measure	Age-standardized rate (per 100,000)						% Change (1990 to 2019)		
		1990			2019					
		Both	Female	Male	Both	Female	Male	Both	Female	Male
Alborz	Incidence	9.52 (6.47 to 13.13)	4.39 (3 to 6.86)	14.38 (9.46 to 20.74)	10.95 (9.16 to 12.88)	7.34 (5.83 to 9.19)	14.42 (11.55 to 17.73)	15.1 (-20.5 to 71.9)	67 (4.5 to 170)	0.3 (-35.7 to 62.5)
	Prevalence	9.25 (6.16 to 12.85)	4.45 (3.03 to 6.89)	13.67 (8.68 to 19.84)	11.4 (9.6 to 13.4)	8.54 (6.83 to 10.72)	14.18 (11.35 to 17.49)	23.2 (-15 to 87)	91.7 (19.1 to 226.9)	3.8 (-34.1 to 72.1)
	Deaths	10.2 (6.99 to 13.67)	4.65 (3.08 to 6.93)	15.55 (10.3 to 21.74)	11.49 (9.74 to 13.55)	7.32 (5.83 to 9.01)	15.45 (12.51 to 19)	12.7 (-19.8 to 75)	57.3 (-3.2 to 157.7)	-0.7 (-33.4 to 58.5)
	DALYs	233.23 (154.11 to 315.31)	110.17 (66.93 to 168.07)	345.96 (219.19 to 489.41)	246.49 (208.9 to 290.87)	153.9 (123.72 to 189.02)	336.47 (273.46 to 418.7)	5.7 (-25.5 to 68.2)	39.7 (-15.3 to 139.1)	-2.7 (-36.6 to 62.3)
	YLLs	231.07 (152.64 to 311.55)	109.12 (66.07 to 166.8)	342.76 (216.19 to 485.73)	243.99 (206.31 to 288.29)	152.18 (122.06 to 187.31)	333.23 (269.92 to 415.11)	5.6 (-25.5 to 67.7)	39.5 (-15.4 to 138.6)	-2.8 (-36.7 to 62.2)
	YLDs	2.16 (1.23 to 3.27)	1.05 (0.58 to 1.69)	3.2 (1.75 to 5.06)	2.49 (1.72 to 3.44)	1.72 (1.14 to 2.4)	3.24 (2.08 to 4.67)	15.4 (-24.4 to 85.9)	63.4 (-0.7 to 177.9)	1.3 (-40.3 to 76.5)
Ardebil	Incidence	11.76 (8.98 to 15.46)	4.88 (3.67 to 6.87)	17.63 (12.77 to 24.15)	16.06 (13.95 to 18.3)	8.86 (7.34 to 10.48)	23.76 (19.93 to 28.05)	36.5 (0.5 to 89.1)	81.5 (17.4 to 161.6)	34.7 (-7.4 to 97.8)
	Prevalence	11.53 (8.69 to 15.3)	4.98 (3.67 to 7.05)	17.04 (12.14 to 23.6)	16.32 (14.15 to 18.7)	9.87 (8.12 to 11.73)	23.19 (19.39 to 27.67)	41.5 (3.4 to 98)	98.3 (28.6 to 182.2)	36.1 (-8.9 to 105.1)
	Deaths	12.5 (9.35 to 16.44)	5.12 (3.76 to 7.11)	18.88 (13.55 to 25.94)	16.92 (14.61 to 19.38)	8.9 (7.38 to 10.45)	25.52 (21.38 to 30)	35.4 (-2.5 to 92.4)	73.8 (10.8 to 144.3)	35.1 (-7.7 to 103.4)
	DALYs	298.67 (222.69 to 393.31)	128.99 (94.79 to 181.31)	441.71 (307.36 to 612.69)	382.65 (329.55 to 439.07)	205.72 (170.02 to 244.47)	570.06 (470.7 to 680.44)	28.1 (-8.6 to 84)	59.5 (0.1 to 127.5)	29.1 (-13.7 to 102.4)
	YLLs	296.01 (220.39 to 390.4)	127.83 (93.62 to 180.09)	437.78 (303.48 to 608.17)	379.06 (326.31 to 434.67)	203.63 (167.85 to 241.93)	564.87 (465.98 to 674.9)	28.1 (-8.7 to 84.3)	59.3 (0.2 to 128.2)	29 (-13.7 to 102.6)
	YLDs	2.66 (1.65 to 3.89)	1.16 (0.72 to 1.77)	3.93 (2.38 to 5.93)	3.59 (2.44 to 4.88)	2.09 (1.39 to 2.88)	5.2 (3.41 to 7.16)	35 (-6.8 to 95.4)	79.6 (10.9 to 169.2)	32.2 (-15 to 110.9)

Province	Measure	Age-standardized rate (per 100,000)						% Change (1990 to 2019)		
		1990			2019					
		Both	Female	Male	Both	Female	Male	Both	Female	Male
Bushehr	Incidence	13.47 (10.16 to 17.05)	6.29 (4.46 to 9.06)	20.53 (14.9 to 26.86)	16.41 (14.21 to 19.06)	11.5 (9.55 to 13.57)	21.51 (18.12 to 25.59)	21.8 (-11.5 to 72.6)	82.8 (16 to 173.6)	4.8 (-26.9 to 54.8)
	Prevalence	12.88 (9.65 to 16.37)	6.35 (4.4 to 9.21)	19.13 (13.68 to 24.98)	16.72 (14.47 to 19.6)	13.12 (10.92 to 15.64)	20.47 (17.19 to 24.55)	29.8 (-5.5 to 87.2)	106.6 (29 to 218.2)	7 (-25.4 to 62.2)
	Deaths	14.48 (10.94 to 18.5)	6.68 (4.79 to 9.83)	22.3 (16.13 to 29.14)	17.35 (15.04 to 19.82)	11.45 (9.78 to 13.41)	23.5 (19.68 to 27.85)	19.8 (-12.6 to 67.1)	71.3 (7.1 to 152.5)	5.4 (-26.3 to 54.7)
	DALYs	330.71 (248.68 to 425.18)	161.57 (114.82 to 238.84)	491.82 (351.55 to 651.97)	375.74 (324.01 to 432.14)	258.77 (222.26 to 301.94)	495.64 (411.2 to 592.66)	13.6 (-18.6 to 62.8)	60.2 (-0.5 to 143.2)	0.8 (-31.1 to 52.6)
	YLLs	327.69 (246.1 to 421.87)	160.09 (113.24 to 237.19)	487.3 (346.69 to 645.29)	372.05 (320.79 to 429.02)	256.07 (219.62 to 298.92)	490.92 (408 to 587.4)	13.5 (-18.8 to 62.6)	60 (-1 to 143.6)	0.7 (-31.2 to 52.9)
	YLDs	3.02 (1.86 to 4.27)	1.48 (0.85 to 2.3)	4.52 (2.7 to 6.56)	3.69 (2.51 to 4.91)	2.7 (1.81 to 3.77)	4.72 (3.16 to 6.52)	22.1 (-12.5 to 81.2)	82.3 (9 to 189.6)	4.3 (-29.6 to 65.1)
Chahar Mahaal and Bakhtiari	Incidence	10.21 (7.96 to 13.08)	4.61 (3.43 to 5.97)	15.4 (11.46 to 20.63)	11.02 (8.87 to 13.59)	6.63 (5.14 to 8.83)	15.69 (11.75 to 19.93)	8 (-25.4 to 57.1)	43.9 (-3.7 to 123.4)	1.9 (-36.3 to 55.6)
	Prevalence	10 (7.74 to 12.93)	4.71 (3.48 to 6.14)	14.71 (10.78 to 19.87)	11.48 (9.25 to 14.27)	7.9 (6.12 to 10.63)	15.27 (11.35 to 19.56)	14.8 (-21.4 to 68.6)	67.7 (11.2 to 156.6)	3.8 (-36.4 to 61.3)
	Deaths	10.9 (8.46 to 13.99)	4.87 (3.62 to 6.34)	16.64 (12.24 to 22.28)	11.56 (9.2 to 14.25)	6.47 (4.89 to 8.49)	17 (12.65 to 21.49)	6.1 (-28.1 to 53.5)	32.8 (-13.7 to 104.5)	2.2 (-36.8 to 56.5)
	DALYs	253.53 (193.95 to 326.71)	117.63 (86.89 to 155)	373.99 (273.35 to 509.3)	254.27 (202.61 to 315.07)	148.68 (112.81 to 194.95)	365.13 (267.6 to 464.12)	0.3 (-32.5 to 48.4)	26.4 (-18.5 to 97.8)	-2.4 (-41.1 to 54.1)
	YLLs	251.2 (191.92 to 324.57)	116.52 (85.79 to 153.61)	370.55 (270.34 to 505.14)	251.74 (200.15 to 311.86)	147.06 (111.3 to 192.78)	361.62 (265.05 to 460.42)	0.2 (-32.6 to 48.7)	26.2 (-18.7 to 97.5)	-2.4 (-41.4 to 54.4)
	YLDs	2.33 (1.53 to 3.43)	1.11 (0.69 to 1.65)	3.44 (2.13 to 5.19)	2.53 (1.63 to 3.56)	1.61 (0.99 to 2.35)	3.5 (2.16 to 5.12)	8.8 (-28.9 to 65.4)	45.5 (-9.4 to 128.2)	1.9 (-42.5 to 67.8)

Province	Measure	Age-standardized rate (per 100,000)						% Change (1990 to 2019)		
		1990			2019					
		Both	Female	Male	Both	Female	Male	Both	Female	Male
East Azarbayejan	Incidence	12.92 (9.6 to 16.37)	6.05 (4.45 to 8.86)	19.12 (13.3 to 25.23)	16.79 (14.09 to 19.9)	11.08 (8.86 to 13.6)	22.63 (18.37 to 27.83)	30 (-4.9 to 85.6)	83.3 (11.9 to 169.1)	18.4 (-18.2 to 82.7)
	Prevalence	12.4 (9.14 to 15.84)	5.97 (4.33 to 8.82)	18.11 (12.41 to 24.03)	16.66 (14 to 19.78)	11.92 (9.53 to 14.77)	21.51 (17.31 to 26.61)	34.3 (-1.7 to 93.3)	99.5 (20.3 to 193.8)	18.8 (-18.4 to 86.6)
	Deaths	13.94 (10.24 to 17.8)	6.51 (4.63 to 9.58)	20.75 (14.68 to 27.25)	18.03 (15.35 to 21.32)	11.45 (9.07 to 14.13)	24.75 (20.07 to 30.29)	29.3 (-6 to 84.3)	75.7 (7.1 to 170.4)	19.3 (-18.2 to 80.8)
	DALYs	318.27 (232.88 to 412.39)	152.16 (108.99 to 222.23)	465.51 (323.99 to 619.81)	383.16 (325.97 to 452.52)	244.48 (195.98 to 301.65)	524.49 (421.18 to 647.32)	20.4 (-13.5 to 74)	60.7 (0.8 to 144.1)	12.7 (-25.7 to 76.6)
	YLLs	315.36 (230.44 to 409.75)	150.75 (107.81 to 220.71)	461.26 (319.93 to 615.26)	379.44 (322.17 to 448.73)	241.97 (193.45 to 298.8)	519.53 (416.7 to 642.55)	20.3 (-13.5 to 73.9)	60.5 (0.4 to 144.1)	12.6 (-25.8 to 76.8)
	YLDs	2.91 (1.84 to 4.17)	1.41 (0.86 to 2.17)	4.25 (2.51 to 6.32)	3.72 (2.57 to 5.02)	2.51 (1.64 to 3.55)	4.96 (3.24 to 6.99)	27.7 (-11.2 to 87.5)	77.8 (7.7 to 174.8)	16.5 (-24.7 to 89)
Fars	Incidence	10.13 (7.53 to 13.42)	3.97 (2.83 to 5.62)	16.35 (11.61 to 22.35)	11.86 (9.84 to 14.13)	7.19 (5.76 to 8.72)	16.53 (13.06 to 20.88)	17 (-18.2 to 69.5)	81.2 (18.1 to 177.8)	1.1 (-33.4 to 60.3)
	Prevalence	9.89 (7.29 to 13.09)	4.02 (2.78 to 5.72)	15.56 (10.89 to 21.4)	12.3 (10.25 to 14.7)	8.27 (6.6 to 10.11)	16.34 (12.78 to 20.76)	24.4 (-13.1 to 83.2)	105.7 (31.3 to 220.4)	5 (-30.9 to 68.2)
	Deaths	10.84 (8.08 to 14.34)	4.22 (3.02 to 5.87)	17.69 (12.45 to 24.13)	12.48 (10.4 to 14.89)	7.19 (5.66 to 8.84)	17.73 (13.94 to 22.18)	15.1 (-21.4 to 67.9)	70.4 (8.7 to 164.6)	0.2 (-36.3 to 58.6)
	DALYs	251.78 (185.47 to 338.14)	100.4 (70.24 to 140.49)	397.11 (275.35 to 547.88)	280.07 (233.55 to 334.02)	162.64 (127.79 to 199.68)	397.74 (311.2 to 502.31)	11.2 (-26 to 67.4)	62 (1.5 to 153.9)	0.2 (-38.4 to 61.5)
	YLLs	249.49 (183.49 to 335.08)	99.46 (69.42 to 139.36)	393.49 (271.5 to 543.18)	277.37 (230.97 to 331.31)	160.94 (125.97 to 198.06)	394.02 (307.31 to 498.62)	11.2 (-26.2 to 67.3)	61.8 (1.5 to 153.7)	0.1 (-38.5 to 61.9)
	YLDs	2.29 (1.37 to 3.38)	0.95 (0.55 to 1.46)	3.62 (2.02 to 5.53)	2.71 (1.81 to 3.71)	1.7 (1.09 to 2.43)	3.72 (2.39 to 5.31)	18.2 (-20.7 to 81.1)	79.4 (12.3 to 198.1)	2.7 (-36.9 to 76.5)

Province	Measure	Age-standardized rate (per 100,000)						% Change (1990 to 2019)		
		1990			2019					
		Both	Female	Male	Both	Female	Male	Both	Female	Male
Gilan	Incidence	10.88 (8.31 to 14)	3.89 (2.78 to 5.63)	18.74 (13.48 to 24.59)	12.83 (10.84 to 15.17)	7.09 (5.72 to 8.6)	18.77 (15.07 to 23.09)	17.9 (-15 to 65.8)	82 (13.9 to 175.3)	0.2 (-32.1 to 49.3)
	Prevalence	10.36 (7.86 to 13.28)	3.84 (2.74 to 5.62)	17.3 (12.37 to 22.8)	13.37 (11.35 to 15.76)	8.19 (6.56 to 10)	18.73 (14.88 to 23.09)	29.1 (-7 to 80.3)	113.2 (32.8 to 226.2)	8.3 (-26.9 to 65.1)
	Deaths	11.51 (8.68 to 14.65)	4.11 (2.97 to 5.92)	20.11 (14.49 to 26.18)	13.37 (11.23 to 15.75)	7.04 (5.75 to 8.57)	19.9 (15.97 to 24.36)	16.2 (-15.4 to 66.4)	71.3 (5.2 to 157.9)	-1.1 (-31.2 to 53.3)
	DALYs	276.81 (209.6 to 353.77)	99.14 (70.45 to 143.85)	463.79 (327.11 to 608)	307.1 (257.28 to 364.68)	161.58 (132.18 to 197.66)	457.54 (364.26 to 570.39)	10.9 (-20.7 to 59.1)	63 (1 to 150.2)	-1.3 (-33.3 to 53.3)
	YLLs	274.34 (207.25 to 351.12)	98.21 (69.44 to 142.17)	459.64 (323.52 to 604.32)	304.17 (254.36 to 361.45)	159.9 (130.78 to 195.94)	453.31 (360.68 to 565.93)	10.9 (-20.9 to 59.1)	62.8 (0.7 to 149.7)	-1.4 (-33.5 to 53.9)
	YLDs	2.47 (1.57 to 3.51)	0.93 (0.57 to 1.49)	4.16 (2.46 to 5.99)	2.93 (2.02 to 3.95)	1.68 (1.11 to 2.41)	4.23 (2.75 to 5.91)	18.7 (-18.8 to 73.6)	80 (3.7 to 183.4)	1.7 (-36.4 to 64.8)
Golestan	Incidence	10.95 (8.22 to 13.8)	4.46 (3.25 to 6.48)	17.27 (12.46 to 22.21)	13.21 (11.48 to 15.12)	7.64 (6.24 to 9.27)	19.18 (15.96 to 22.95)	20.6 (-10.1 to 71.7)	71.2 (6.9 to 161.9)	11 (-19.6 to 66.8)
	Prevalence	10.61 (7.95 to 13.42)	4.44 (3.21 to 6.55)	16.38 (11.73 to 21.34)	13.5 (11.75 to 15.48)	8.43 (6.86 to 10.27)	18.93 (15.72 to 22.73)	27.2 (-4.9 to 82.4)	89.8 (16.8 to 192)	15.5 (-16.4 to 71.9)
	Deaths	11.65 (8.71 to 14.93)	4.73 (3.51 to 6.74)	18.59 (13.37 to 24.17)	13.8 (11.96 to 15.84)	7.67 (6.33 to 9.27)	20.36 (16.89 to 24.42)	18.4 (-11 to 65)	62.3 (-2.6 to 137.5)	9.5 (-20.5 to 61.6)
	DALYs	278.88 (211 to 360.85)	117.57 (86.21 to 169.54)	429.09 (308.08 to 569.11)	324.84 (281.54 to 374.86)	185.5 (150.22 to 224.4)	473.72 (392.01 to 565.58)	16.5 (-14.7 to 66.5)	57.8 (-6.8 to 136.7)	10.4 (-21.8 to 65.8)
	YLLs	276.36 (209.11 to 357.06)	116.5 (84.87 to 168.12)	425.2 (305.88 to 564.25)	321.83 (279.19 to 371.41)	183.7 (148.75 to 222.33)	469.42 (388.37 to 559.8)	16.5 (-14.9 to 66.4)	57.7 (-7 to 136.9)	10.4 (-21.8 to 66.1)
	YLDs	2.51 (1.59 to 3.6)	1.08 (0.65 to 1.69)	3.89 (2.39 to 5.71)	3.01 (2.04 to 4.12)	1.8 (1.17 to 2.57)	4.3 (2.79 to 6.08)	19.7 (-13.5 to 73.6)	67.3 (-0.2 to 168.1)	10.7 (-24.8 to 73.7)

Province	Measure	Age-standardized rate (per 100,000)						% Change (1990 to 2019)		
		1990			2019					
		Both	Female	Male	Both	Female	Male	Both	Female	Male
Hamadan	Incidence	10.29 (7.61 to 13.7)	3.79 (2.79 to 5.26)	16.24 (11.34 to 22.68)	12.53 (10.4 to 14.78)	6.59 (5.4 to 7.9)	18.68 (14.93 to 22.95)	21.7 (-17 to 75.4)	73.6 (11.9 to 155.3)	15 (-26.8 to 82.8)
	Prevalence	10.29 (7.64 to 13.85)	3.86 (2.84 to 5.5)	16.12 (11.16 to 22.49)	12.99 (10.84 to 15.33)	7.35 (5.99 to 8.96)	18.82 (14.97 to 23.15)	26.3 (-13.5 to 82.6)	90.4 (23 to 175)	16.8 (-26 to 87.8)
	Deaths	10.85 (8.19 to 14.68)	4.02 (3.02 to 5.64)	17.15 (12.24 to 23.79)	13.09 (10.83 to 15.42)	6.68 (5.41 to 8.1)	19.7 (15.76 to 24.26)	20.6 (-20 to 71.6)	66.3 (7 to 139)	14.9 (-27.3 to 76.6)
	DALYs	265.38 (201 to 356.38)	98.32 (73.26 to 138.56)	417.2 (296.47 to 580.51)	308.49 (255.37 to 363.95)	153.4 (124.54 to 186.26)	468.39 (370.5 to 574.47)	16.2 (-23.4 to 67.5)	56 (1.8 to 125.4)	12.3 (-29.7 to 74.3)
	YLLs	262.99 (198.79 to 353.31)	97.4 (72.48 to 137.67)	413.48 (293.1 to 574.97)	305.59 (252.31 to 360.85)	151.83 (123.12 to 184.75)	464.12 (366.05 to 570.61)	16.2 (-23.5 to 68)	55.9 (1.4 to 126.3)	12.2 (-29.8 to 75.3)
	YLDs	2.39 (1.48 to 3.57)	0.92 (0.58 to 1.41)	3.72 (2.22 to 5.72)	2.9 (1.92 to 4.01)	1.57 (1.07 to 2.22)	4.27 (2.69 to 6.12)	21.4 (-19.5 to 86.4)	70.4 (5.7 to 156.7)	14.6 (-31.1 to 96.6)
Hormozgan	Incidence	8.33 (5.75 to 11.18)	3.13 (2.16 to 4.57)	13.05 (8.75 to 17.83)	9.4 (7.83 to 11.57)	5.67 (4.57 to 6.87)	13.28 (10.43 to 17.1)	12.9 (-23 to 75.7)	81.2 (11.1 to 183.4)	1.8 (-36.2 to 69.4)
	Prevalence	8.05 (5.39 to 10.83)	3.11 (2.09 to 4.6)	12.36 (8.09 to 17.13)	9.66 (8.12 to 11.89)	6.18 (4.97 to 7.51)	13.28 (10.41 to 17.13)	19.9 (-18.6 to 89.5)	99.1 (18.6 to 216.4)	7.4 (-34 to 80.3)
	Deaths	8.88 (6.25 to 11.92)	3.31 (2.38 to 4.83)	14.09 (9.6 to 19.29)	9.84 (8.15 to 12.02)	5.77 (4.65 to 6.99)	14.06 (10.95 to 18.2)	10.7 (-24.8 to 69.2)	74.3 (2 to 158.1)	-0.2 (-37.1 to 63.7)
	DALYs	212.85 (140.61 to 286.91)	82.57 (54.67 to 124.49)	325.78 (211.84 to 458.64)	230.22 (190.2 to 281.36)	132.74 (106.06 to 161.76)	331.19 (257.13 to 430.16)	8.2 (-28.3 to 74.9)	60.8 (-9.1 to 154.7)	1.7 (-37.4 to 73.3)
	YLLs	210.92 (139.15 to 284.66)	81.79 (54.11 to 123.36)	322.83 (209.27 to 454.17)	228.04 (188.35 to 279.63)	131.38 (104.73 to 160.6)	328.14 (254.47 to 425.32)	8.1 (-28.3 to 74.6)	60.6 (-9.1 to 155.3)	1.6 (-37.4 to 73.6)
	YLDs	1.93 (1.11 to 2.9)	0.78 (0.44 to 1.22)	2.95 (1.65 to 4.57)	2.18 (1.45 to 3.1)	1.36 (0.89 to 1.9)	3.05 (1.9 to 4.6)	13.5 (-28.6 to 88.5)	74.4 (5.1 to 175.1)	3.2 (-42 to 88.6)

Province	Measure	Age-standardized rate (per 100,000)						% Change (1990 to 2019)		
		1990			2019					
		Both	Female	Male	Both	Female	Male	Both	Female	Male
Ilam	Incidence	10.26 (7.58 to 13.67)	4.08 (2.67 to 6.37)	14.96 (10.67 to 20.11)	13.09 (11.21 to 15.04)	8.73 (7.11 to 10.41)	17.17 (14.13 to 20.78)	27.6 (-9.7 to 83.1)	114.2 (23.2 to 240.2)	14.8 (-22.3 to 77.2)
	Prevalence	9.94 (7.29 to 13.12)	4.11 (2.66 to 6.44)	14.28 (10.06 to 19.2)	13.47 (11.54 to 15.47)	9.99 (8.13 to 11.99)	16.8 (13.77 to 20.41)	35.6 (-5.1 to 96.1)	143 (37.6 to 290.5)	17.7 (-21.2 to 84.8)
	Deaths	11 (8.06 to 14.61)	4.32 (2.89 to 6.67)	16.14 (11.63 to 21.79)	13.83 (11.97 to 15.98)	8.67 (6.89 to 10.25)	18.56 (15.38 to 22.34)	25.8 (-12.4 to 82.5)	100.8 (10.1 to 219.4)	15 (-23.6 to 76.8)
	DALYs	255.71 (187.16 to 342.79)	105.03 (68.04 to 158.51)	368.49 (256.62 to 502.69)	305.88 (261.89 to 351.53)	201.21 (160.92 to 240.21)	407.45 (334.37 to 496.74)	19.6 (-17.3 to 75.7)	91.6 (5.4 to 213.6)	10.6 (-27.6 to 74.4)
	YLLs	253.38 (184.96 to 339.8)	104.05 (67.21 to 157.16)	365.13 (254.2 to 498.02)	302.9 (259.67 to 348.5)	199.13 (159.58 to 238.09)	403.62 (330.86 to 493.26)	19.5 (-17.4 to 75.5)	91.4 (5 to 214.5)	10.5 (-27.8 to 74.5)
	YLDs	2.34 (1.47 to 3.48)	0.99 (0.55 to 1.65)	3.36 (2.01 to 5.03)	2.98 (2.05 to 4.04)	2.08 (1.34 to 2.89)	3.83 (2.54 to 5.34)	27.5 (-14.6 to 92.1)	111 (13.7 to 253.9)	14.1 (-28.3 to 88.3)
Isfahan	Incidence	9.51 (6.9 to 12.63)	4.6 (3.2 to 6.94)	14.64 (10 to 20.08)	12.14 (10.09 to 14.26)	8.49 (6.84 to 10.41)	15.72 (12.48 to 19.3)	27.7 (-9 to 89)	84.5 (12.3 to 196.5)	7.3 (-27.5 to 71.6)
	Prevalence	9.33 (6.72 to 12.44)	4.63 (3.18 to 6.99)	14.04 (9.56 to 19.33)	12.53 (10.46 to 14.7)	9.54 (7.63 to 11.73)	15.47 (12.18 to 18.99)	34.3 (-5 to 98.9)	105.9 (25.3 to 243.2)	10.2 (-26.2 to 77.8)
	Deaths	10.2 (7.2 to 13.63)	4.95 (3.42 to 7.33)	15.84 (10.73 to 21.69)	12.8 (10.83 to 15.03)	8.6 (6.93 to 10.61)	16.89 (13.68 to 20.62)	25.5 (-11.9 to 84.7)	74 (5.1 to 177.1)	6.6 (-30.5 to 69.7)
	DALYs	233.07 (167.5 to 310.99)	112.1 (74.05 to 167.15)	353.11 (236.33 to 487.05)	278.54 (235.62 to 330.92)	182.75 (148.09 to 227.16)	372.56 (298.28 to 460.29)	19.5 (-16.4 to 79.5)	63 (-0.2 to 169.5)	5.5 (-31.2 to 70.9)
	YLLs	230.91 (165.56 to 308.72)	111.01 (73.24 to 165.59)	349.86 (233.55 to 483.54)	275.77 (233.17 to 328.09)	180.76 (146.2 to 225.49)	369.02 (295.86 to 455.9)	19.4 (-16.6 to 79.8)	62.8 (-0.2 to 169.8)	5.5 (-31.4 to 71.2)
	YLDs	2.16 (1.32 to 3.19)	1.1 (0.64 to 1.76)	3.25 (1.89 to 5.06)	2.77 (1.86 to 3.81)	1.99 (1.29 to 2.82)	3.53 (2.24 to 5.09)	28.4 (-13.9 to 95)	81.6 (4 to 203.6)	8.9 (-34 to 89.5)

Province	Measure	Age-standardized rate (per 100,000)						% Change (1990 to 2019)		
		1990			2019					
		Both	Female	Male	Both	Female	Male	Both	Female	Male
Kerman	Incidence	15.48 (11.82 to 19.79)	6.5 (4.93 to 8.92)	24.13 (17.43 to 31.86)	16.18 (13.6 to 19.17)	9.87 (8.02 to 11.9)	22.48 (18.06 to 28.02)	4.5 (-24.9 to 50.4)	51.9 (-1.1 to 115.1)	-6.8 (-36 to 46.1)
	Prevalence	14.9 (11.34 to 19.17)	6.38 (4.79 to 8.84)	22.84 (16.37 to 30.34)	16.3 (13.7 to 19.43)	10.52 (8.47 to 12.7)	22.09 (17.8 to 27.51)	9.4 (-22 to 58.3)	64.9 (7.4 to 132.3)	-3.3 (-34.6 to 56)
	Deaths	16.57 (12.57 to 21.2)	6.98 (5.25 to 9.64)	26.02 (18.96 to 34.54)	17.2 (14.52 to 20.16)	10.23 (8.38 to 12.29)	24.15 (19.53 to 29.2)	3.8 (-24.3 to 46.9)	46.5 (-5.7 to 108.5)	-7.2 (-36.8 to 39.1)
	DALYs	389.31 (295.48 to 505.54)	164.92 (125.15 to 228.99)	597.19 (433.33 to 807.32)	388.04 (327.76 to 456.82)	226.21 (184.83 to 272.73)	549.65 (442.06 to 672.05)	-0.3 (-28.5 to 41.2)	37.2 (-11.8 to 93.8)	-8 (-39 to 41)
	YLLs	385.88 (292.58 to 501.79)	163.4 (123.78 to 227.85)	591.96 (428.57 to 801.73)	384.42 (324.62 to 452.8)	223.94 (182.83 to 270.68)	544.67 (438.13 to 667.15)	-0.4 (-28.8 to 41.3)	37.1 (-11.9 to 94.1)	-8 (-39.3 to 41)
	YLDs	3.43 (2.1 to 4.97)	1.52 (0.93 to 2.35)	5.24 (3.1 to 7.77)	3.63 (2.5 to 4.96)	2.27 (1.49 to 3.19)	4.99 (3.33 to 7.06)	5.8 (-27.8 to 63)	49 (-7.4 to 125.4)	-4.8 (-39.7 to 61.2)
Kermanshah	Incidence	14.87 (10.96 to 19.71)	5.53 (4.03 to 7.7)	22.52 (15.66 to 30.8)	13.82 (11.54 to 16.22)	8.51 (6.86 to 10.38)	19.26 (15.13 to 23.68)	-7.1 (-34.6 to 34.5)	53.8 (-2.1 to 128.8)	-14.5 (-44.5 to 32.6)
	Prevalence	14.32 (10.47 to 19.08)	5.49 (3.94 to 7.73)	21.44 (14.82 to 29.61)	14.14 (11.79 to 16.61)	9.35 (7.51 to 11.37)	19.11 (14.96 to 23.74)	-1.3 (-30.4 to 45.8)	70.2 (8.4 to 156.2)	-10.9 (-43 to 40.9)
	Deaths	15.89 (11.69 to 20.87)	5.88 (4.29 to 8.24)	24.19 (16.96 to 32.67)	14.53 (12.09 to 17.18)	8.63 (6.99 to 10.42)	20.52 (16.39 to 25.21)	-8.6 (-36.7 to 36.1)	46.7 (-6.5 to 119.7)	-15.2 (-45.4 to 35.1)
	DALYs	373.72 (275.75 to 488.38)	143.05 (101.46 to 203.16)	560.78 (388.13 to 761.15)	334.3 (276.5 to 394.71)	198.72 (159.94 to 240.8)	475.21 (374.21 to 589.78)	-10.5 (-38.1 to 34.7)	38.9 (-13.2 to 109.8)	-15.3 (-46.4 to 38.6)
	YLLs	370.4 (273.42 to 485.04)	141.75 (100.34 to 201.24)	555.82 (383.32 to 756.42)	331.15 (273.89 to 391.45)	196.72 (157.96 to 238.66)	470.87 (369.58 to 585.96)	-10.6 (-38.2 to 34.6)	38.8 (-13.3 to 110.4)	-15.3 (-46.5 to 38.5)
	YLDs	3.32 (2.05 to 4.84)	1.3 (0.8 to 2.02)	4.97 (2.89 to 7.47)	3.15 (2.17 to 4.27)	2 (1.32 to 2.85)	4.33 (2.81 to 6.19)	-5.1 (-36.7 to 41.2)	53.9 (-6 to 139.1)	-12.7 (-46 to 42.2)

Province	Measure	Age-standardized rate (per 100,000)						% Change (1990 to 2019)		
		1990			2019					
		Both	Female	Male	Both	Female	Male	Both	Female	Male
Khorasan-e-Razavi	Incidence	13.51 (10.36 to 17.49)	6.82 (5.03 to 8.85)	19.67 (14.17 to 26.84)	14.81 (12.62 to 17.33)	11.01 (9.05 to 13.4)	18.76 (15.32 to 22.96)	9.6 (-21.9 to 52.4)	61.4 (14.7 to 137.6)	-4.6 (-36.4 to 46.8)
	Prevalence	13.04 (9.94 to 16.98)	6.7 (4.84 to 8.82)	18.76 (13.39 to 25.74)	15.1 (12.86 to 17.64)	11.96 (9.84 to 14.57)	18.35 (14.92 to 22.53)	15.7 (-18.2 to 62.4)	78.5 (26 to 162.8)	-2.2 (-35.1 to 53.1)
	Deaths	14.44 (11.09 to 18.48)	7.3 (5.46 to 9.42)	21.12 (15.47 to 28.64)	15.63 (13.44 to 18.31)	11.25 (9.18 to 13.52)	20.18 (16.67 to 24.82)	8.2 (-22.2 to 50.6)	54.1 (5.6 to 123.7)	-4.5 (-36.8 to 43.5)
	DALYs	339.22 (260.29 to 438.73)	173.92 (126.56 to 229.05)	488.09 (353.59 to 670.55)	350.06 (299.56 to 409.6)	250.97 (204.17 to 302.13)	452.83 (370.58 to 554.02)	3.2 (-25.4 to 43.7)	44.3 (-1.5 to 110.2)	-7.2 (-39 to 44.1)
	YLLs	336.19 (257.97 to 435.32)	172.35 (125.48 to 227.19)	483.74 (349.88 to 665.1)	346.74 (296.5 to 405.82)	248.44 (202.3 to 299.3)	448.68 (366.89 to 549.85)	3.1 (-25.4 to 43.8)	44.1 (-1.6 to 110.7)	-7.2 (-39.1 to 44.2)
	YLDs	3.02 (1.9 to 4.31)	1.57 (0.95 to 2.32)	4.35 (2.55 to 6.45)	3.32 (2.21 to 4.53)	2.53 (1.66 to 3.48)	4.15 (2.76 to 5.83)	9.9 (-25.6 to 60.7)	61.4 (8.1 to 159.8)	-4.6 (-41.1 to 57.3)
Khuzestan	Incidence	10.11 (7.72 to 13.26)	4.28 (3.12 to 6.15)	16.02 (11.65 to 21.41)	11.92 (10.16 to 14.03)	7.68 (6.11 to 9.44)	16.29 (13.2 to 20.06)	17.9 (-14.6 to 65.4)	79.5 (14.1 to 167.1)	1.7 (-30.3 to 55.4)
	Prevalence	9.88 (7.56 to 13.03)	4.3 (3.14 to 6.26)	15.27 (11.07 to 20.67)	12.24 (10.47 to 14.41)	8.56 (6.82 to 10.53)	16.01 (12.9 to 19.86)	23.9 (-11.2 to 73.4)	99.2 (25.9 to 200.6)	4.8 (-28.9 to 60.4)
	Deaths	10.77 (8.26 to 13.97)	4.52 (3.35 to 6.32)	17.25 (12.63 to 23.3)	12.5 (10.79 to 14.5)	7.74 (6.21 to 9.47)	17.4 (14.37 to 21.25)	16.1 (-19.1 to 60.8)	71.2 (7.4 to 151.3)	0.9 (-32.2 to 49.1)
	DALYs	254.97 (197 to 333.78)	110.08 (80.59 to 154.47)	394.16 (283.55 to 538.57)	285.32 (243.87 to 333.27)	178.57 (142.34 to 220.71)	394.74 (323.33 to 486.59)	11.9 (-22.5 to 55.3)	62.2 (1.6 to 141.4)	0.1 (-34 to 53.3)
	YLLs	252.67 (194.55 to 331.46)	109.05 (79.75 to 153.12)	390.61 (281.24 to 534.95)	282.6 (241.44 to 330.36)	176.76 (140.7 to 219.08)	391.07 (320.05 to 483.53)	11.8 (-22.5 to 55.6)	62.1 (1.4 to 141.4)	0.1 (-34.2 to 53.8)
	YLDs	2.29 (1.46 to 3.36)	1.03 (0.63 to 1.6)	3.55 (2.19 to 5.39)	2.73 (1.85 to 3.74)	1.81 (1.2 to 2.56)	3.67 (2.35 to 5.31)	18.9 (-18.1 to 75)	76 (4.9 to 178.5)	3.4 (-34.2 to 70.1)

Province	Measure	Age-standardized rate (per 100,000)						% Change (1990 to 2019)		
		1990			2019					
		Both	Female	Male	Both	Female	Male	Both	Female	Male
Kohgiluyeh and Boyer-Ahmad	Incidence	8.71 (6.26 to 12.05)	4.03 (2.81 to 6.29)	13.18 (8.64 to 18.92)	10.93 (8.91 to 13.3)	8.3 (6.3 to 10.67)	13.29 (10.09 to 17.4)	25.6 (-18.6 to 91.9)	106 (14.2 to 236.4)	0.8 (-38.8 to 73)
	Prevalence	8.63 (6.14 to 12.02)	4.08 (2.8 to 6.51)	12.77 (8.33 to 18.52)	11.57 (9.35 to 14.02)	9.7 (7.29 to 12.45)	13.3 (10.09 to 17.42)	34 (-12.8 to 103.7)	137.6 (28.8 to 294.6)	4.2 (-37.6 to 79.8)
	Deaths	9.27 (6.55 to 13.01)	4.28 (2.98 to 6.58)	14.18 (9.22 to 21.09)	11.43 (9.27 to 13.89)	8.27 (6.21 to 10.69)	14.2 (10.73 to 18.58)	23.3 (-19.2 to 85.7)	93.1 (7.8 to 209.3)	0.2 (-39.9 to 71.3)
	DALYs	222.1 (155.83 to 313.7)	104.31 (70.8 to 158.11)	327.98 (210.14 to 495.7)	259.69 (212.27 to 318.01)	191.83 (144.41 to 249.75)	322.79 (242.44 to 422.14)	16.9 (-23.8 to 77.3)	83.9 (3.1 to 199.6)	-1.6 (-41.6 to 72.8)
	YLLs	220.11 (154.34 to 311.43)	103.34 (69.91 to 156.97)	325.03 (207.95 to 491.13)	257.17 (209.36 to 315.59)	189.85 (142.8 to 247.91)	319.78 (239.99 to 419.24)	16.8 (-23.9 to 77.4)	83.7 (2.9 to 200.2)	-1.6 (-41.7 to 73.3)
	YLDs	1.99 (1.2 to 2.99)	0.97 (0.57 to 1.61)	2.95 (1.62 to 4.69)	2.52 (1.65 to 3.54)	1.98 (1.18 to 2.87)	3.02 (1.86 to 4.48)	26.9 (-19.5 to 107.4)	105 (12.5 to 242.4)	2.3 (-43.1 to 94.6)
Kurdistan	Incidence	15.53 (11.76 to 20.31)	5.6 (4.28 to 7.61)	23.65 (16.85 to 31.87)	14.66 (12.29 to 17.35)	8.85 (7.25 to 10.53)	20.5 (16.12 to 25.64)	-5.6 (-33.9 to 32.4)	57.9 (7 to 120.8)	-13.3 (-44.3 to 31.5)
	Prevalence	15.04 (11.24 to 19.76)	5.66 (4.33 to 7.62)	22.66 (15.98 to 30.88)	14.93 (12.57 to 17.67)	9.65 (7.86 to 11.56)	20.31 (15.89 to 25.61)	-0.7 (-31.3 to 41.2)	70.3 (12.6 to 141)	-10.4 (-43 to 38.7)
	Deaths	16.63 (12.62 to 21.75)	5.93 (4.43 to 8.34)	25.47 (18.68 to 34.34)	15.47 (13.08 to 18.19)	9.03 (7.52 to 10.73)	21.88 (17.39 to 26.99)	-7 (-35.9 to 31.2)	52.4 (-2.5 to 117.1)	-14.1 (-44 to 29.9)
	DALYs	388.71 (291.27 to 511.88)	146.51 (110.06 to 205.13)	586.42 (420.54 to 801)	350.61 (295.28 to 417.26)	203.57 (167.89 to 243.45)	500.08 (397.82 to 626.73)	-9.8 (-38.2 to 30.3)	38.9 (-13.8 to 99)	-14.7 (-45.8 to 33.1)
	YLLs	385.22 (288.11 to 507.96)	145.18 (108.63 to 203.23)	581.17 (416.19 to 795.53)	347.27 (291.77 to 413.14)	201.5 (165.47 to 241.53)	495.47 (392.73 to 621.06)	-9.9 (-38.2 to 30.5)	38.8 (-14 to 98.9)	-14.7 (-46 to 33.2)
	YLDs	3.49 (2.26 to 5.23)	1.33 (0.85 to 2)	5.25 (3.14 to 8.21)	3.33 (2.27 to 4.63)	2.07 (1.39 to 2.88)	4.61 (2.98 to 6.7)	-4.6 (-36.6 to 42.3)	55.8 (-1.7 to 136.2)	-12.3 (-47.3 to 44.4)

Province	Measure	Age-standardized rate (per 100,000)						% Change (1990 to 2019)		
		1990			2019					
		Both	Female	Male	Both	Female	Male	Both	Female	Male
Lorestan	Incidence	10.92 (8.21 to 14.41)	4.45 (3.29 to 6.19)	16.55 (11.88 to 22.6)	13.02 (10.69 to 15.86)	7.7 (5.95 to 9.56)	18.63 (14.78 to 23.5)	19.2 (-19.5 to 71)	73.3 (9.2 to 158.6)	12.6 (-29.7 to 72.3)
	Prevalence	10.53 (7.92 to 13.87)	4.48 (3.3 to 6.25)	15.63 (11.1 to 21.69)	13.35 (10.9 to 16.11)	8.84 (6.7 to 10.91)	18.17 (14.3 to 23.08)	26.8 (-14.4 to 80.9)	97.1 (20.3 to 195.8)	16.2 (-28.6 to 79.2)
	Deaths	11.73 (8.76 to 15.58)	4.7 (3.39 to 6.38)	17.96 (12.8 to 24.48)	13.73 (11.52 to 16.61)	7.65 (5.96 to 9.55)	20.11 (16.36 to 25.32)	17 (-19.8 to 71.5)	62.9 (4.4 to 147.6)	12 (-27.1 to 71.8)
	DALYs	270.61 (200.45 to 362.61)	115.32 (83.97 to 156.95)	401.46 (281.09 to 560.68)	306.66 (254.33 to 374.02)	178.56 (137.3 to 225.92)	442.66 (354.47 to 562.05)	13.3 (-22.5 to 68.6)	54.8 (-3.2 to 138.8)	10.3 (-30 to 74.5)
	YLLs	268.17 (198.23 to 359.29)	114.25 (82.86 to 155.54)	397.84 (277.95 to 556.55)	303.7 (251.7 to 370.14)	176.73 (135.22 to 224)	438.5 (350.71 to 557.65)	13.2 (-22.7 to 68.2)	54.7 (-3.4 to 138.8)	10.2 (-30.1 to 74.5)
	YLDs	2.45 (1.53 to 3.57)	1.07 (0.66 to 1.65)	3.63 (2.18 to 5.54)	2.96 (1.98 to 4.1)	1.83 (1.18 to 2.64)	4.16 (2.66 to 6)	21.1 (-21.4 to 84.7)	71 (3.6 to 164.2)	14.6 (-32.5 to 88.9)
Markazi	Incidence	11.81 (9.07 to 15.51)	5.27 (3.91 to 7.19)	18.11 (12.95 to 25.05)	10.95 (9.18 to 12.88)	7.3 (5.86 to 8.82)	14.71 (11.48 to 18.36)	-7.3 (-35.8 to 32.1)	38.5 (-10.5 to 100.1)	-18.7 (-47.8 to 24.9)
	Prevalence	11.64 (8.86 to 15.33)	5.31 (3.94 to 7.21)	17.66 (12.6 to 24.58)	11.43 (9.54 to 13.5)	8.14 (6.52 to 9.86)	14.82 (11.53 to 18.58)	-1.7 (-32.1 to 40.7)	53.3 (-1.4 to 124.8)	-16.1 (-46.5 to 31.2)
	Deaths	12.57 (9.57 to 16.52)	5.61 (4.14 to 7.47)	19.33 (13.7 to 26.72)	11.45 (9.68 to 13.43)	7.41 (6.01 to 8.93)	15.61 (12.53 to 19.28)	-8.9 (-36.5 to 27.8)	32 (-13.2 to 91.2)	-19.3 (-47.7 to 23.6)
	DALYs	300.07 (226.11 to 394.97)	135.78 (101 to 182.58)	456.85 (322.88 to 642.76)	265.46 (225.1 to 316.02)	168.21 (136.29 to 202.51)	365.29 (289.41 to 453.97)	-11.5 (-38.7 to 27.4)	23.9 (-18.5 to 81)	-20 (-49 to 26)
	YLLs	297.4 (224.05 to 392.05)	134.54 (99.67 to 180.8)	452.83 (319.91 to 638.19)	262.93 (222.54 to 312.4)	166.49 (134.55 to 200.74)	361.91 (286.23 to 450.23)	-11.6 (-38.6 to 27.6)	23.7 (-18.6 to 81.2)	-20.1 (-49.3 to 26.2)
	YLDs	2.66 (1.67 to 3.93)	1.24 (0.76 to 1.86)	4.03 (2.42 to 6.24)	2.54 (1.66 to 3.47)	1.72 (1.12 to 2.43)	3.38 (2.14 to 4.84)	-4.7 (-36.5 to 44.3)	38.8 (-12.9 to 120.9)	-16 (-49.5 to 40)

Province	Measure	Age-standardized rate (per 100,000)						% Change (1990 to 2019)		
		1990			2019					
		Both	Female	Male	Both	Female	Male	Both	Female	Male
Mazandaran	Incidence	8.39 (6.16 to 11.19)	3.22 (2.22 to 5.26)	13.83 (9.67 to 18.55)	10.57 (8.86 to 12.38)	6.39 (5.19 to 7.87)	14.89 (11.82 to 18.2)	25.9 (-10.9 to 82.4)	98.1 (7.3 to 213.5)	7.7 (-27.5 to 65.4)
	Prevalence	8.27 (6.08 to 11.03)	3.33 (2.27 to 5.49)	13.25 (9.19 to 17.83)	11.21 (9.42 to 13.16)	7.68 (6.15 to 9.41)	14.87 (11.75 to 18.3)	35.5 (-4.3 to 96.6)	130.2 (26.8 to 266.7)	12.2 (-25.6 to 76.1)
	Deaths	8.95 (6.56 to 11.84)	3.38 (2.33 to 5.69)	14.96 (10.39 to 20.32)	10.96 (9.32 to 12.98)	6.22 (5.09 to 7.61)	15.86 (12.86 to 19.48)	22.5 (-11.2 to 76)	83.9 (1.5 to 184)	6.1 (-27.5 to 64)
	DALYs	207.06 (154.04 to 276.3)	80.98 (55.14 to 135.12)	333.26 (228.55 to 457.82)	247.6 (210.6 to 293.2)	143.02 (117.19 to 175.92)	355.74 (287.41 to 437.51)	19.6 (-14.6 to 73)	76.6 (-1 to 177.1)	6.7 (-28.9 to 68)
	YLLs	205.14 (152.66 to 273.83)	80.19 (54.45 to 134.02)	330.16 (226.64 to 454.45)	245.17 (208.6 to 290.17)	141.5 (115.88 to 174.25)	352.36 (284.46 to 434.75)	19.5 (-14.4 to 72.9)	76.5 (-1.4 to 177.1)	6.7 (-29.2 to 68.2)
	YLDs	1.93 (1.16 to 2.88)	0.79 (0.45 to 1.35)	3.1 (1.76 to 4.75)	2.43 (1.64 to 3.36)	1.52 (0.99 to 2.18)	3.37 (2.18 to 4.79)	26.1 (-16.4 to 90.2)	92.1 (0.3 to 225.8)	8.9 (-33.7 to 82.5)
North Khorasan	Incidence	10.27 (7.56 to 13.8)	4.3 (3.22 to 5.61)	15.64 (10.89 to 22.24)	11.5 (9.88 to 13.12)	7.85 (6.37 to 9.49)	15.23 (12.52 to 18.05)	12 (-22.8 to 57)	82.6 (26.8 to 163.2)	-2.6 (-38.3 to 47.7)
	Prevalence	9.99 (7.29 to 13.47)	4.24 (3.18 to 5.54)	15.07 (10.48 to 21.57)	11.65 (10.07 to 13.44)	8.43 (6.89 to 10.2)	15.01 (12.32 to 17.88)	16.6 (-19.6 to 65.4)	98.9 (36.3 to 191.5)	-0.4 (-36.8 to 54.8)
	Deaths	10.88 (8.15 to 14.49)	4.56 (3.36 to 6.24)	16.66 (11.59 to 23.16)	12.08 (10.43 to 13.9)	8.01 (6.46 to 9.81)	16.2 (13.34 to 19.04)	11 (-23.6 to 55.2)	75.4 (18.6 to 157.7)	-2.8 (-36.9 to 49.4)
	DALYs	262.11 (193.67 to 353.49)	111.72 (83.3 to 152.65)	395.01 (273.16 to 550.96)	278.63 (241.25 to 320.34)	184.96 (150.25 to 224.74)	376.04 (307.14 to 447.28)	6.3 (-26.6 to 50)	65.6 (11.1 to 139.8)	-4.8 (-38.5 to 50.5)
	YLLs	259.76 (191.5 to 350.09)	110.69 (82.47 to 151.28)	391.49 (270 to 546.49)	276 (239.01 to 317.77)	183.13 (148.45 to 222.69)	372.61 (304.13 to 443.41)	6.3 (-26.6 to 50.1)	65.4 (10.8 to 140.1)	-4.8 (-38.6 to 50.6)
	YLDs	2.34 (1.42 to 3.58)	1.03 (0.65 to 1.51)	3.52 (2.02 to 5.59)	2.63 (1.74 to 3.54)	1.84 (1.14 to 2.61)	3.44 (2.2 to 4.88)	12 (-27.1 to 67.5)	78.5 (13.2 to 182.6)	-2.5 (-42.5 to 61)

Province	Measure	Age-standardized rate (per 100,000)						% Change (1990 to 2019)		
		1990			2019					
		Both	Female	Male	Both	Female	Male	Both	Female	Male
Qazvin	Incidence	9.25 (6.84 to 11.84)	3.29 (2.38 to 4.73)	15.15 (10.64 to 19.86)	11.77 (9.83 to 14.02)	5.79 (4.58 to 7.29)	18.19 (14.89 to 21.85)	27.2 (-9.1 to 83.2)	75.9 (4.9 to 171.6)	20 (-16.6 to 80.9)
	Prevalence	8.94 (6.52 to 11.46)	3.27 (2.34 to 4.71)	14.34 (9.97 to 18.66)	11.81 (9.91 to 14.11)	6.47 (5.08 to 8.18)	17.47 (14.36 to 21.06)	32 (-5.4 to 89.4)	98 (18.7 to 201)	21.8 (-15.9 to 84.4)
	Deaths	9.86 (7.31 to 12.62)	3.51 (2.48 to 5.05)	16.34 (11.83 to 21.33)	12.49 (10.47 to 14.88)	5.81 (4.56 to 7.17)	19.74 (16.15 to 23.89)	26.7 (-7.8 to 86.1)	65.6 (4.1 to 151.4)	20.8 (-15.6 to 83)
	DALYs	231.32 (169.49 to 298.45)	84.84 (59.54 to 121.17)	369.48 (264.01 to 491.14)	274.91 (231.38 to 325.61)	132.31 (105.32 to 163.17)	425.09 (349.45 to 518.34)	18.8 (-14.4 to 74.2)	56 (-1.9 to 138.4)	15 (-20.9 to 75)
	YLLs	229.21 (168.14 to 295.79)	84.03 (58.98 to 120.12)	366.11 (260.64 to 487.42)	272.24 (228.8 to 322.38)	130.92 (103.96 to 161.37)	421.04 (346.06 to 513.87)	18.8 (-14.6 to 74.5)	55.8 (-2.2 to 138.7)	15 (-21.1 to 75.1)
	YLDs	2.11 (1.31 to 3.05)	0.81 (0.48 to 1.24)	3.37 (2.04 to 5.06)	2.67 (1.85 to 3.76)	1.39 (0.92 to 1.98)	4.04 (2.67 to 5.87)	26.8 (-14.3 to 95.1)	71.9 (0.2 to 175.7)	20 (-24 to 96.6)
Qom	Incidence	12.62 (9.25 to 16.71)	6.09 (4.16 to 8.71)	18.99 (13.04 to 25.93)	11.86 (10.09 to 13.81)	8.54 (7.05 to 10.24)	14.96 (11.97 to 18.27)	-6.1 (-35.4 to 39)	40.3 (-11.2 to 118.8)	-21.2 (-49.3 to 25)
	Prevalence	12.14 (8.61 to 16.09)	5.9 (3.8 to 8.35)	18.07 (12.24 to 24.94)	11.97 (10.17 to 13.95)	9.12 (7.52 to 10.98)	14.66 (11.72 to 18.08)	-1.4 (-32 to 45.9)	54.7 (-1.9 to 152.3)	-18.9 (-47.8 to 29.3)
	Deaths	13.55 (10.11 to 17.51)	6.57 (4.51 to 9.65)	20.45 (14.18 to 27.5)	12.59 (10.71 to 14.73)	8.84 (7.23 to 10.62)	16.06 (12.98 to 19.63)	-7.1 (-33.6 to 32.9)	34.6 (-14.2 to 108.8)	-21.5 (-46.9 to 19.3)
	DALYs	314.55 (223.96 to 412.44)	152.24 (95.02 to 222.38)	468.01 (321.94 to 637.82)	276.81 (235.33 to 323.45)	188.23 (153.77 to 226.24)	360.74 (289.96 to 441.07)	-12 (-37.6 to 28.6)	23.6 (-20.9 to 100.4)	-22.9 (-48.4 to 20.5)
	YLLs	311.75 (222.71 to 407.69)	150.85 (94.19 to 220.64)	463.85 (318.07 to 632.84)	274.12 (233.03 to 321.03)	186.25 (152.05 to 223.95)	357.38 (286.79 to 437.57)	-12.1 (-37.7 to 28.8)	23.5 (-21 to 100.8)	-23 (-48.5 to 20.6)
	YLDs	2.8 (1.69 to 4.1)	1.39 (0.78 to 2.17)	4.16 (2.39 to 6.28)	2.69 (1.81 to 3.6)	1.98 (1.29 to 2.77)	3.36 (2.18 to 4.76)	-4.1 (-35.7 to 47.3)	41.8 (-13.5 to 139.9)	-19.2 (-49.9 to 37.1)

Province	Measure	Age-standardized rate (per 100,000)						% Change (1990 to 2019)		
		1990			2019					
		Both	Female	Male	Both	Female	Male	Both	Female	Male
Semnan	Incidence	11.33 (8.62 to 14.67)	4.53 (3.13 to 7.21)	18.73 (13.52 to 24.92)	12.46 (10.51 to 14.73)	7.38 (5.81 to 9.06)	17.89 (14.44 to 21.54)	10 (-20.5 to 54.9)	63 (-5.6 to 157.5)	-4.5 (-35.4 to 40.7)
	Prevalence	10.98 (8.19 to 14.31)	4.55 (3.13 to 7.24)	17.66 (12.63 to 23.71)	12.72 (10.78 to 15.06)	8.43 (6.62 to 10.41)	17.24 (13.98 to 20.81)	15.9 (-16.4 to 64.2)	85.1 (6 to 196.2)	-2.4 (-34.5 to 43.6)
	Deaths	12.1 (9.03 to 15.75)	4.82 (3.35 to 7.47)	20.29 (14.59 to 27.11)	13.18 (11.09 to 15.6)	7.37 (5.87 to 9.03)	19.47 (15.73 to 23.63)	8.9 (-24.6 to 55.8)	52.8 (-10.9 to 135.9)	-4 (-35.8 to 44.9)
	DALYs	282.66 (207.87 to 373.75)	115.54 (80.61 to 179.2)	454.15 (324.98 to 615.82)	288.81 (243.66 to 339.16)	166.71 (132.19 to 204.52)	415.94 (334.24 to 502.13)	2.2 (-28.6 to 47.4)	44.3 (-14.3 to 124.5)	-8.4 (-38.9 to 38.8)
	YLLs	280.1 (205.75 to 371.41)	114.46 (79.71 to 177.8)	450.03 (321.71 to 609.97)	286 (241.81 to 336.43)	164.96 (130.17 to 202.52)	412 (331.12 to 497.78)	2.1 (-28.9 to 47.3)	44.1 (-14.6 to 124.7)	-8.5 (-39 to 39)
	YLDs	2.56 (1.58 to 3.71)	1.08 (0.61 to 1.85)	4.12 (2.5 to 6.1)	2.81 (1.89 to 3.89)	1.75 (1.13 to 2.49)	3.94 (2.54 to 5.7)	10.1 (-23.9 to 61.5)	61.3 (-9.7 to 168.6)	-4.4 (-38.1 to 50)
Sistan and Baluchistan	Incidence	9.33 (6.04 to 12.67)	3.88 (2.75 to 5.15)	13.63 (8.62 to 19.27)	10.54 (8.65 to 12.56)	7.04 (5.47 to 8.92)	13.96 (10.94 to 17.42)	12.9 (-23.5 to 79.3)	81.6 (20.4 to 176.5)	2.4 (-35.1 to 73)
	Prevalence	9.08 (5.74 to 12.45)	3.91 (2.59 to 5.26)	13.08 (8.03 to 18.66)	10.58 (8.7 to 12.67)	7.53 (5.86 to 9.58)	13.6 (10.57 to 17.04)	16.5 (-22.1 to 90.1)	92.6 (26.6 to 202.4)	4 (-35.3 to 81.5)
	Deaths	9.93 (6.7 to 13.27)	4.08 (2.97 to 5.48)	14.63 (9.26 to 20.65)	11.09 (9.23 to 13.27)	7.17 (5.55 to 9.18)	14.91 (11.65 to 18.7)	11.7 (-22.6 to 72.9)	75.6 (9.1 to 165.9)	1.9 (-35.6 to 68.6)
	DALYs	240.04 (149.58 to 323.03)	104.5 (70.47 to 143.62)	344.41 (203.08 to 490.83)	264.59 (220.53 to 314.85)	181.12 (141.71 to 229.6)	347.33 (270.3 to 441.84)	10.2 (-25 to 78.1)	73.3 (6.7 to 175.5)	0.8 (-36.2 to 74.6)
	YLLs	237.89 (148.06 to 320.16)	103.55 (69.57 to 142.12)	341.32 (200.84 to 487.37)	262.17 (217.95 to 312.26)	179.46 (140.24 to 227.46)	344.16 (267.79 to 438.02)	10.2 (-25.1 to 78.1)	73.3 (6.9 to 176.3)	0.8 (-36.3 to 75.2)
	YLDs	2.15 (1.21 to 3.24)	0.95 (0.58 to 1.4)	3.09 (1.67 to 4.87)	2.42 (1.63 to 3.38)	1.66 (1.06 to 2.4)	3.16 (1.99 to 4.66)	12.3 (-28.5 to 89.9)	73.9 (8.5 to 180.9)	2.2 (-40.7 to 89.4)

Province	Measure	Age-standardized rate (per 100,000)						% Change (1990 to 2019)		
		1990			2019					
		Both	Female	Male	Both	Female	Male	Both	Female	Male
South Khorasan	Incidence	10.37 (7.92 to 13.82)	4.23 (3.15 to 5.73)	15.99 (11.35 to 22.16)	11.44 (9.71 to 13.37)	7.81 (6.29 to 9.58)	15.39 (12.58 to 18.53)	10.3 (-24.6 to 54.8)	84.8 (25.4 to 169.5)	-3.7 (-37.1 to 46.5)
	Prevalence	10.13 (7.71 to 13.52)	4.26 (3.21 to 5.8)	15.39 (10.93 to 21.54)	11.65 (9.88 to 13.58)	8.61 (6.95 to 10.57)	14.96 (12.12 to 18.14)	15 (-22.2 to 60.9)	102.1 (34.1 to 190.6)	-2.8 (-38.2 to 48.3)
	Deaths	11.09 (8.29 to 14.92)	4.47 (3.35 to 6)	17.22 (12.19 to 24.14)	12.12 (10.35 to 14.11)	7.92 (6.35 to 9.76)	16.7 (13.47 to 20.27)	9.3 (-25.9 to 55.8)	77 (17.7 to 150.3)	-3 (-37 to 50.1)
	DALYs	261.61 (194.22 to 352.18)	110.12 (83.41 to 147.3)	397.15 (274.94 to 563.46)	272 (231.39 to 317.07)	183.07 (148.15 to 224.76)	367.8 (293.31 to 449.95)	4 (-29 to 50.3)	66.2 (10.3 to 137)	-7.4 (-41.5 to 48.7)
	YLLs	259.25 (192.51 to 349.53)	109.1 (82.46 to 146.25)	393.58 (272.52 to 559.21)	269.4 (228.7 to 315.2)	181.24 (146.44 to 222.99)	364.36 (290.25 to 446.73)	3.9 (-29 to 50.6)	66.1 (10.3 to 137.1)	-7.4 (-41.6 to 48.7)
	YLDs	2.36 (1.47 to 3.44)	1.02 (0.62 to 1.48)	3.57 (2.15 to 5.46)	2.6 (1.78 to 3.54)	1.82 (1.16 to 2.56)	3.44 (2.28 to 4.81)	10.2 (-28.3 to 63.4)	78.8 (14.9 to 176.8)	-3.6 (-42.2 to 60.2)
Tehran	Incidence	8.32 (6.19 to 11.67)	3.94 (2.83 to 5.88)	12.85 (8.8 to 19.11)	8.33 (6.95 to 9.82)	6.14 (4.83 to 7.71)	10.5 (8.27 to 13.1)	0.1 (-32.5 to 41.2)	55.9 (-1.1 to 133.8)	-18.3 (-49.5 to 29.5)
	Prevalence	8.22 (6.08 to 11.63)	4 (2.87 to 6.02)	12.42 (8.47 to 18.52)	8.5 (7.06 to 10.05)	6.78 (5.35 to 8.58)	10.22 (8.02 to 12.76)	3.4 (-31.1 to 46.9)	69.3 (6.7 to 154.6)	-17.8 (-49.3 to 32.1)
	Deaths	8.9 (6.48 to 12.33)	4.22 (3 to 6.53)	13.83 (9.31 to 20.36)	8.74 (7.29 to 10.32)	6.23 (4.94 to 7.69)	11.22 (8.89 to 13.99)	-1.8 (-33 to 38.9)	47.6 (-9.8 to 121.1)	-18.8 (-47.8 to 32.1)
	DALYs	201.06 (146.71 to 279.47)	93.49 (67.54 to 147.22)	307.34 (207.5 to 458.81)	185.06 (155.4 to 218.49)	126.8 (100.73 to 159.17)	243.23 (192.9 to 304.89)	-8 (-37.4 to 31.7)	35.6 (-16.7 to 102.9)	-20.9 (-49.7 to 30.2)
	YLLs	199.15 (145.3 to 276.56)	92.55 (66.65 to 145.7)	304.43 (205.28 to 454.58)	183.14 (153.63 to 216.74)	125.36 (99.24 to 157.35)	240.84 (190.22 to 302.29)	-8 (-37.5 to 32.2)	35.4 (-17.1 to 103.2)	-20.9 (-50 to 30.7)
	YLDs	1.91 (1.15 to 2.93)	0.94 (0.58 to 1.53)	2.91 (1.6 to 4.66)	1.91 (1.27 to 2.63)	1.44 (0.89 to 2.06)	2.39 (1.52 to 3.43)	0 (-33.6 to 51.6)	52.5 (-7.5 to 143.1)	-17.9 (-50.6 to 43.1)

Province	Measure	Age-standardized rate (per 100,000)						% Change (1990 to 2019)		
		1990			2019					
		Both	Female	Male	Both	Female	Male	Both	Female	Male
West Azarbayejan	Incidence	18.12 (13.81 to 23.31)	6.95 (5.2 to 9.75)	28.58 (20.84 to 38)	19.11 (16.36 to 21.96)	10.79 (8.72 to 13.01)	28.05 (23.06 to 33.11)	5.5 (-23.2 to 48.5)	55.2 (-1.7 to 124.6)	-1.9 (-30.1 to 47.3)
	Prevalence	17.09 (12.98 to 22.04)	6.85 (5.11 to 9.7)	26.4 (19.1 to 35.14)	18.74 (16.02 to 21.52)	11.52 (9.33 to 13.96)	26.44 (21.78 to 31.39)	9.7 (-20.2 to 53.5)	68.2 (4 to 144.1)	0.2 (-29 to 52.5)
	Deaths	19.68 (15.23 to 25.48)	7.49 (5.6 to 10.65)	31.38 (23.28 to 41.77)	20.63 (17.7 to 23.96)	11.23 (9.07 to 13.62)	30.8 (25.85 to 36.68)	4.9 (-23.1 to 44.3)	49.9 (-2.1 to 118.7)	-1.9 (-31.5 to 42.2)
	DALYs	439.28 (338.71 to 570.53)	176.16 (131.38 to 251.25)	677.37 (493.46 to 916.07)	438.91 (377.36 to 507.13)	242.89 (199.18 to 290.61)	646.74 (537.86 to 777.64)	-0.1 (-28 to 40.4)	37.9 (-10.4 to 100.4)	-4.5 (-34.6 to 42.3)
	YLLs	435.3 (333.74 to 566.27)	174.56 (130.08 to 249.39)	671.18 (489.64 to 910.65)	434.69 (373.48 to 502.24)	240.41 (196.91 to 288.28)	640.65 (533.26 to 770.93)	-0.1 (-28.1 to 40.1)	37.7 (-10.3 to 100.2)	-4.5 (-34.7 to 42.8)
	YLDs	3.98 (2.53 to 5.67)	1.59 (0.98 to 2.41)	6.2 (3.81 to 9.14)	4.22 (2.88 to 5.8)	2.47 (1.6 to 3.44)	6.08 (4.07 to 8.46)	5.8 (-26.2 to 57.8)	55.3 (-6.5 to 138.5)	-1.8 (-35.6 to 57.4)
Yazd	Incidence	12.18 (9.13 to 15.42)	5.83 (4.21 to 8.43)	19.55 (14 to 25.49)	13.66 (11.22 to 16.4)	9.25 (7.38 to 11.33)	18.12 (14.1 to 22.83)	12.2 (-20.7 to 59.6)	58.6 (-1.8 to 140)	-7.3 (-38 to 44.1)
	Prevalence	11.82 (8.84 to 15.08)	5.78 (4.18 to 8.44)	18.42 (12.92 to 24.34)	13.98 (11.48 to 16.76)	10.51 (8.35 to 13.02)	17.41 (13.48 to 22.07)	18.4 (-16 to 71.6)	81.8 (12.4 to 174.6)	-5.5 (-37.4 to 49.1)
	Deaths	13.02 (9.75 to 16.59)	6.24 (4.43 to 9.04)	21.19 (15.43 to 27.39)	14.45 (11.82 to 17.31)	9.29 (7.39 to 11.35)	19.79 (15.28 to 24.8)	10.9 (-20.3 to 57.3)	48.7 (-8.6 to 128.5)	-6.6 (-38.4 to 45.7)
	DALYs	301.86 (225.84 to 385.03)	146.52 (103.79 to 211.73)	469.09 (334.04 to 614.88)	309.7 (254.54 to 370.45)	200.95 (160.67 to 245.71)	415.71 (320.97 to 523.22)	2.6 (-26.9 to 46.3)	37.2 (-15.3 to 112.3)	-11.4 (-42.9 to 39.3)
	YLLs	299.14 (223.61 to 381.76)	145.16 (102.78 to 209.72)	464.83 (330.67 to 610.48)	306.63 (251.8 to 367.11)	198.78 (158.48 to 243.63)	411.72 (318.25 to 517.27)	2.5 (-27 to 46.7)	36.9 (-15.6 to 112.2)	-11.4 (-43.2 to 39.4)
	YLDs	2.72 (1.71 to 3.91)	1.36 (0.8 to 2.12)	4.26 (2.49 to 6.24)	3.08 (2.1 to 4.32)	2.17 (1.4 to 3.12)	3.98 (2.56 to 5.86)	12.9 (-23.6 to 70.9)	59.4 (-7.6 to 153)	-6.5 (-43.3 to 56)

Province	Measure	Age-standardized rate (per 100,000)						% Change (1990 to 2019)		
		1990			2019					
		Both	Female	Male	Both	Female	Male	Both	Female	Male
Zanjan	Incidence	10.92 (8.33 to 14.7)	4.18 (3 to 7.77)	17.52 (12.94 to 23.95)	11.65 (10.05 to 13.34)	6.87 (5.78 to 8.21)	16.85 (13.87 to 20.01)	6.7 (-22.4 to 48.7)	64.6 (-15.8 to 145.4)	-3.8 (-31.6 to 37.9)
	Prevalence	10.59 (8.07 to 14.33)	4.19 (2.99 to 7.7)	16.56 (12.22 to 22.77)	11.77 (10.11 to 13.43)	7.56 (6.33 to 9.01)	16.33 (13.43 to 19.57)	11.1 (-20 to 54.4)	80.4 (-8.7 to 169.2)	-1.4 (-31.1 to 44)
	Deaths	11.61 (8.76 to 15.54)	4.43 (3.14 to 7.56)	18.9 (13.69 to 25.5)	12.33 (10.51 to 14.21)	6.95 (5.76 to 8.26)	18.22 (14.9 to 21.59)	6.2 (-22.7 to 47.6)	56.9 (-17.2 to 132.7)	-3.6 (-30.1 to 41.8)
	DALYs	275.73 (208.33 to 368.33)	108.36 (75.67 to 183.36)	430.41 (308.34 to 587.51)	272.15 (231.22 to 316.09)	155.03 (128.59 to 183.43)	398.37 (323.57 to 479.57)	-1.3 (-29.2 to 37.5)	43.1 (-24.5 to 113.6)	-7.4 (-34.6 to 38.9)
	YLLs	273.25 (206.18 to 365.61)	107.35 (74.91 to 181.4)	426.52 (304.93 to 582.78)	269.51 (229.06 to 313.23)	153.41 (127.24 to 181.4)	394.61 (320.26 to 474.87)	-1.4 (-29.4 to 37.5)	42.9 (-24.4 to 113.9)	-7.5 (-34.8 to 38.8)
	YLDs	2.48 (1.54 to 3.65)	1.01 (0.59 to 1.79)	3.89 (2.39 to 5.85)	2.64 (1.82 to 3.63)	1.62 (1.06 to 2.32)	3.76 (2.43 to 5.29)	6.6 (-25.7 to 52.9)	60.6 (-21.5 to 157.7)	-3.4 (-35.3 to 46.6)

Data in parentheses are 95% Uncertainty Intervals (95% UIs)

DALYs=Disability-Adjusted Life Years; YLLs=Years of Life Lost; YLDs=Years Lived with Disability
