# Supplementary material for: Estimates of incidence, prevalence, mortality, and disability‐adjusted life years of lung cancer in Iran, 1990–2019: A systematic analysis from the global burden of disease study 2019
Source: Cancer Med. 2022 Jun 13;11(23):4624–40. doi: 10.1002/cam4.4792 (PMC9741968; doi:10.1002/cam4.4792)
Supplement: Supplementary file 5 — Table S2 [file CAM4-11-4624-s004.pdf]

Location		Sex	New cases		Expected new cases in 2019		% 1990 - 2019 new cases change cause			% 1990 - 2019 new cases overall change
			1990	2019	Population growth	Population growth + Aging	Population growth	Age structure change	Incidence rate change	
Iran (Islamic Republic of)		Both	2865	8705	4126	8001	44.0%	135.2%	24.6%	203.8%
		Female	590	2827	855	1741	44.9%	150.3%	184.0%	379.2%
		Male	2275	5878	3256	6119	43.1%	125.8%	-10.6%	158.3%
Sub-national	Alborz	Both	52	261	102	236	95.4%	255.2%	47.4%	398.0%
		Female	12	84	24	54	99.0%	259.9%	247.4%	606.3%
		Male	41	177	78	178	92.0%	247.5%	-2.3%	337.2%
	Ardebil	Both	58	180	65	134	10.9%	119.4%	78.3%	208.5%
		Female	12	52	13	29	10.9%	142.4%	194.2%	347.5%
		Male	47	128	52	98	10.8%	97.6%	65.9%	174.3%
	Bushehr	Both	35	133	61	113	71.4%	147.6%	57.2%	276.2%
		Female	8	48	14	27	64.7%	159.7%	244.5%	468.9%
		Male	27	85	48	84	78.0%	133.9%	4.3%	216.2%
	Chahar Mahaal and Bakhtiari	Both	30	88	41	82	35.3%	137.5%	18.3%	191.1%
		Female	6	28	9	19	36.0%	164.7%	125.2%	325.9%
		Male	24	60	32	60	34.8%	119.1%	0.7%	154.5%
	East Azarbayejan	Both	206	621	244	493	18.2%	120.9%	61.6%	200.7%
		Female	46	206	54	117	18.1%	137.1%	194.8%	350.1%
		Male	161	415	190	361	18.2%	106.3%	33.7%	158.3%
	Fars	Both	160	513	218	444	35.9%	141.0%	42.7%	219.7%
		Female	30	155	41	88	36.5%	153.5%	221.4%	411.4%
		Male	130	358	176	352	35.4%	135.5%	4.1%	175.0%
	Gilan	Both	139	393	155	339	11.6%	132.0%	39.1%	182.7%
		Female	24	109	27	61	11.7%	140.3%	201.2%	353.2%
		Male	115	284	128	285	11.4%	136.3%	-0.9%	146.9%
	Golestan	Both	60	195	86	163	43.9%	127.9%	53.9%	225.8%
		Female	12	59	17	35	44.3%	146.0%	199.0%	389.2%
		Male	48	136	69	123	43.5%	112.2%	28.9%	184.7%
	Hamadan	Both	86	220	89	181	3.4%	107.1%	44.8%	155.3%
		Female	15	58	15	34	5.1%	124.8%	163.4%	293.4%
		Male	71	162	73	141	1.8%	95.8%	29.2%	126.9%

Location	Sex	New cases		Expected new cases in 2019		% 1990 - 2019 new cases change cause			% 1990 - 2019 new cases overall change
		1990	2019	Population growth	Population growth + Aging	Populatio n growth	Age structure change	Incidence rate change	
Hormozgan	Both	33	108	68	97	105.0%	86.8%	35.5%	227.3%
	Female	6	33	12	19	105.1%	124.8%	237.6%	467.6%
	Male	27	76	56	74	104.9%	65.2%	6.4%	176.5%
Ilam	Both	17	59	22	47	32.0%	153.0%	72.7%	257.7%
	Female	3	20	4	10	33.7%	191.6%	351%	576.2%
	Male	14	39	18	34	30.4%	121.7%	36.5%	188.7%
Isfahan	Both	171	614	235	489	37.1%	148.3%	73.1%	258.4%
	Female	40	209	56	116	39.7%	151.4%	232.7%	423.8%
	Male	131	405	177	379	34.6%	153.7%	19.8%	208.2%
Kerman	Both	130	393	232	381	78.3%	114.4%	8.8%	201.4%
	Female	26	118	45	81	76.0%	140.6%	146.6%	363.2%
	Male	105	274	189	295	80.5%	101.6%	-20.1%	162.0%
Kermanshah	Both	110	252	128	274	16.3%	133.2%	-19.6%	130.0%
	Female	19	78	22	52	18.7%	159.2%	139.0%	317.0%
	Male	91	174	104	203	14.1%	109.1%	-31.7%	91.5%
Khorasan-e-Razavi	Both	295	780	418	723	41.4%	103.2%	19.4%	163.9%
	Female	70	295	99	188	41.7%	127.9%	152.1%	321.7%
	Male	226	485	318	513	41.1%	86.2%	-12.2%	115.1%
Khuzestan	Both	128	417	195	359	52.2%	127.8%	45.2%	225.2%
	Female	27	136	41	78	53.2%	136.9%	215.6%	405.7%
	Male	101	281	153	277	51.3%	122.0%	3.9%	177.1%
Kohgiluyeh and Boyer-Ahmad	Both	15	54	23	44	51.9%	142.2%	69.6%	263.8%
	Female	3	20	5	10	51.9%	153.0%	303.6%	508.4%
	Male	12	34	18	34	52.0%	140.2%	2.4%	194.6%
Kurdistan	Both	85	206	113	220	33.8%	126.1%	-16.6%	143.3%
	Female	14	62	19	41	34.2%	149.1%	146.3%	329.6%
	Male	70	144	94	167	33.5%	104.0%	-32.4%	105.1%
Lorestan	Both	67	192	77	162	14.3%	126.8%	44.4%	185.6%
	Female	13	59	15	35	15.5%	153.9%	188.5%	358.0%
	Male	54	132	61	118	13.2%	104.3%	27.0%	144.4%

Location	Sex	New cases		Expected new cases in 2019		% 1990 - 2019 new cases change cause			% 1990 - 2019 new cases overall change
		1990	2019	Population growth	Population growth + Aging	Populatio n growth	Age structure change	Incidence rate change	
Markazi	Both	74	164	88	177	19.2%	120.5%	-16.9%	122.8%
	Female	16	55	19	40	18.5%	138.5%	94.7%	251.8%
	Male	58	109	69	134	19.9%	110.5%	-42.4%	87.9%
Mazandaran	Both	100	380	131	304	31.5%	173.4%	76.6%	281.4%
	Female	19	117	25	60	31.3%	181.7%	297.4%	510.4%
	Male	81	263	106	243	31.6%	170.6%	24.9%	227.1%
North Khorasan	Both	29	81	40	73	37.5%	115.6%	26.8%	179.9%
	Female	6	28	8	16	37.6%	137.6%	214.3%	389.5%
	Male	23	53	32	55	37.3%	99.2%	-7.7%	128.9%
Qazvin	Both	40	129	56	104	39.9%	120.6%	64.9%	225.4%
	Female	7	33	10	19	40.4%	143.9%	205.8%	390.1%
	Male	33	96	46	82	39.3%	108.9%	43.3%	191.5%
Qom	Both	39	121	71	132	84.1%	156.5%	-28.6%	212.0%
	Female	9	41	17	31	85.8%	160.0%	114.7%	360.5%
	Male	30	80	54	101	82.4%	158.8%	-73.7%	167.5%
Semnan	Both	31	82	48	76	54.9%	91.6%	20%	166.5%
	Female	6	25	9	16	57.1%	104.9%	158.6%	320.7%
	Male	25	57	38	61	52.9%	91.8%	-15.4%	129.3%
Sistan and Baluchistan	Both	52	145	106	127	101.9%	40.4%	34.6%	177.0%
	Female	10	51	20	29	102.0%	82.0%	227.0%	411.0%
	Male	42	94	85	92	101.9%	14.5%	5.1%	121.4%
South Khorasan	Both	37	81	46	73	24.2%	72.7%	20.5%	117.4%
	Female	7	29	9	16	24.1%	95.4%	182.3%	301.8%
	Male	30	52	37	55	24.4%	57.1%	-7.7%	73.8%
Tehran	Both	326	1112	534	1138	63.8%	185.0%	-8.0%	240.9%
	Female	75	399	125	265	67.3%	187.5%	180.7%	435.6%
	Male	252	712	404	878	60.5%	188.5%	-65.9%	183.2%
West Azarbayejan	Both	172	492	252	480	46.6%	132.8%	7.1%	186.5%
	Female	32	143	48	97	46.8%	153.3%	142.0%	342.1%
	Male	139	349	204	362	46.5%	113.5%	-9.7%	150.4%

Location		Sex	New cases		Expected new cases in 2019		% 1990 - 2019 new cases change cause			% 1990 - 2019 new cases overall change
			1990	2019	Population growth	Population growth + Aging	Populatio n growth	Age structure change	Incidence rate change	
	Yazd	Both	42	126	71	114	69.2%	100.3%	28.0%	197.5%
		Female	10	43	17	28	70.3%	105.2%	150.3%	325.8%
		Male	32	83	54	91	68.1%	113.8%	-24.3%	157.6%
	Zanjan	Both	45	113	54	107	21.6%	117.9%	13.7%	153.2%
		Female	8	35	10	22	22.1%	144.1%	161.5%	327.7%
		Male	36	78	44	82	21.0%	104.7%	-11.4%	114.3%
