# Supplementary material for: Estimates of incidence, prevalence, mortality, and disability‐adjusted life years of lung cancer in Iran, 1990–2019: A systematic analysis from the global burden of disease study 2019
Source: Cancer Med. 2022 Jun 13;11(23):4624–40. doi: 10.1002/cam4.4792 (PMC9741968; doi:10.1002/cam4.4792)
Supplement: Supplementary file 6 — Table S3 [file CAM4-11-4624-s005.pdf]

Measure	Risk factor	Metric	Year						% Change (1990 to 2019)		
			1990			2019					
			Both	Female	Male	Both	Female	Male	Both	Female	Male
DALYs	Diet low in fruits	Rate	4.7 (1.2 to 7.3)	2.3 (0.6 to 3.8)	7 (1.7 to 11.1)	3.5 (1 to 5.5)	2.4 (0.7 to 3.9)	4.6 (1.4 to 7.4)	-25.9 (-45.9 to 1)	5.1 (-30.3 to 49.4)	-34.6 (-57.7 to -2.9)
		Percent	1.7 (0.5 to 2.6)	1.9 (0.5 to 2.9)	1.7 (0.4 to 2.5)	1.2 (0.4 to 1.9)	1.4 (0.4 to 2.2)	1.2 (0.3 to 1.9)	-28.9 (-41.6 to -9.5)	-29.1 (-45.2 to -9.5)	-30.8 (-48.4 to -7.3)
	High fasting plasma glucose	Rate	14.3 (2.8 to 35)	5.6 (1 to 13.4)	22.4 (3.6 to 57.2)	26.9 (6.2 to 57.8)	17.2 (3.6 to 39.6)	36.9 (6.4 to 84.7)	88.6 (47.3 to 157.2)	204.5 (104.1 to 301.5)	64.5 (26.6 to 125.9)
		Percent	5.2 (1.1 to 11.9)	4.7 (0.9 to 11.3)	5.3 (0.8 to 12.8)	9.4 (2.2 to 20.1)	9.6 (1.9 to 21.9)	9.3 (1.6 to 21.3)	81.5 (65.1 to 121.8)	105.6 (89.3 to 131.4)	74.4 (63.4 to 92)
	Occupational carcinogens	Rate	15 (9.6 to 21.9)	1.6 (1.1 to 2.5)	27 (17.1 to 39.2)	16.7 (11.5 to 22.8)	2.6 (1.7 to 3.5)	30.9 (21.2 to 42.2)	11 (-16.9 to 46.7)	57.2 (2.3 to 118.1)	14.5 (-15.7 to 53.3)
		Percent	5.4 (3.6 to 7.6)	1.4 (0.9 to 1.8)	6.4 (4.3 to 8.8)	5.8 (4 to 7.9)	1.4 (1 to 2)	7.8 (5.4 to 10.5)	6.6 (-7.2 to 24.7)	5.9 (-12 to 29.6)	21.3 (5.5 to 39.2)
	Particulate matter pollution	Rate	62.6 (44.7 to 84.7)	27 (19.4 to 38.4)	95.8 (67.3 to 132.3)	62.7 (47.5 to 79.5)	38.8 (28.5 to 49)	87 (65.1 to 111.4)	0.2 (-21.4 to 28.3)	43.9 (-6.5 to 76.4)	-9.3 (-30.3 to 20.6)
		Percent	22.7 (17.1 to 28.3)	22.5 (16.9 to 28)	22.8 (17.2 to 28.4)	21.9 (16.2 to 27.3)	21.8 (16.2 to 27.1)	21.9 (16.3 to 27.4)	-3.6 (-7.2 to -1.2)	-2.9 (-7.3 to -0.1)	-3.8 (-7.4 to -1.3)
	Residential radon	Rate	13.5 (2.6 to 28.1)	5.9 (1.1 to 12.6)	20.6 (3.9 to 42.6)	14.1 (2.7 to 28.2)	8.7 (1.7 to 17.8)	19.5 (3.8 to 39.6)	4.4 (-18.2 to 33.7)	49 (-3.4 to 83.8)	-5.3 (-27.8 to 27.3)
		Percent	4.9 (1 to 9.9)	4.9 (1 to 10)	4.9 (1 to 9.8)	4.9 (1 to 9.9)	4.9 (1 to 9.9)	4.9 (1 to 9.9)	0.4 (-5.5 to 6.4)	0.2 (-6 to 7.1)	0.6 (-6.2 to 7.5)
	Secondhand smoke	Rate	17.3 (9.8 to 26.7)	9.5 (5.5 to 15.2)	24.4 (13.5 to 38.8)	18.6 (11.1 to 27.8)	14.5 (8.7 to 21.3)	22.9 (13.5 to 34.4)	7.9 (-16.4 to 39)	52.6 (0.3 to 91.3)	-6.5 (-30.1 to 29.1)
		Percent	6.3 (3.8 to 9.2)	7.9 (4.8 to 11.7)	5.8 (3.4 to 8.6)	6.5 (3.9 to 9.4)	8.2 (4.9 to 11.9)	5.8 (3.4 to 8.6)	3.8 (-6.5 to 15.9)	3 (-5.9 to 12.6)	-0.8 (-14.4 to 15.4)
	Smoking	Rate	164.7 (134.3 to 202.2)	21.9 (15.9 to 31.8)	297.7 (239.4 to 366.6)	149.8 (136.9 to 164.5)	31.6 (25.8 to 38)	269.4 (243.9 to 296.9)	-9 (-30.2 to 20.8)	44.4 (-7.2 to 110.9)	-9.5 (-31.3 to 20.4)
		Percent	59.7 (55.7 to 62.9)	18.2 (14.3 to 22.7)	70.7 (68.3 to 73.2)	52.2 (49.8 to 54.7)	17.8 (14.9 to 20.8)	67.9 (65.4 to 70.2)	-12.5 (-17.9 to -5)	-2.6 (-23.9 to 26.6)	-4.1 (-7.4 to -0.6)
YLLs	Diet low in fruits	Rate	4.7 (1.2 to 7.3)	2.3 (0.6 to 3.7)	6.9 (1.7 to 11)	3.5 (1 to 5.4)	2.4 (0.7 to 3.8)	4.5 (1.4 to 7.4)	-26 (-46 to 0.9)	5 (-30.3 to 49)	-34.6 (-57.7 to -2.9)
		Percent	1.7 (0.5 to 2.6)	1.9 (0.5 to 2.9)	1.7 (0.4 to 2.5)	1.2 (0.4 to 1.9)	1.4 (0.4 to 2.2)	1.2 (0.3 to 1.9)	-28.9 (-41.6 to -9.5)	-29.1 (-45.2 to -9.5)	-30.8 (-48.5 to -7.3)
	High fasting plasma glucose	Rate	14.1 (2.7 to 34.7)	5.6 (1 to 13.2)	22.2 (3.6 to 56.6)	26.6 (6.1 to 57.1)	17 (3.6 to 39.2)	36.5 (6.3 to 83.9)	88.4 (47.3 to 157.1)	204.2 (104.1 to 301.5)	64.4 (26.6 to 125.8)
		Percent	5.2 (1.1 to 11.9)	4.7 (0.9 to 11.2)	5.3 (0.8 to 12.8)	9.4 (2.2 to 20.1)	9.6 (1.9 to 21.8)	9.3 (1.6 to 21.3)	81.5 (65.1 to 121.7)	105.6 (89.5 to 131.5)	74.5 (63.3 to 91.9)
	Occupational carcinogens	Rate	14.9 (9.5 to 21.7)	1.6 (1.1 to 2.4)	26.7 (17 to 38.8)	16.5 (11.4 to 22.7)	2.5 (1.7 to 3.5)	30.6 (21 to 41.9)	10.9 (-17 to 46.8)	57.1 (2.2 to 118.2)	14.5 (-15.8 to 53.3)
		Percent	5.5 (3.6 to 7.6)	1.4 (0.9 to 1.8)	6.4 (4.3 to 8.9)	5.8 (4 to 7.9)	1.4 (1 to 2)	7.8 (5.4 to 10.5)	6.7 (-7.2 to 24.8)	5.9 (-12 to 29.7)	21.2 (5.5 to 39.2)

Measure	Risk factor	Metric	Year						% Change (1990 to 2019)		
			1990			2019					
			Both	Female	Male	Both	Female	Male	Both	Female	Male
	Particulate matter pollution	Rate	62.1 (44.2 to 83.9)	26.7 (19.2 to 38)	95 (66.7 to 130.9)	62.1 (47 to 78.5)	38.4 (28.3 to 48.4)	86.1 (64.5 to 110.4)	0.1 (-21.4 to 28.2)	43.7 (-6.5 to 76.3)	-9.3 (-30.4 to 20.5)
		Percent	22.7 (17.1 to 28.3)	22.4 (16.9 to 28)	22.8 (17.2 to 28.4)	21.9 (16.2 to 27.3)	21.8 (16.2 to 27.1)	21.9 (16.3 to 27.4)	-3.6 (-7.2 to -1.2)	-2.9 (-7.3 to -0.1)	-3.8 (-7.4 to -1.3)
	Residential radon	Rate	13.4 (2.6 to 27.8)	5.8 (1.1 to 12.4)	20.4 (3.9 to 42.3)	13.9 (2.7 to 27.9)	8.6 (1.6 to 17.6)	19.3 (3.7 to 39.3)	4.3 (-18.2 to 33.7)	48.8 (-3.6 to 83.6)	-5.3 (-27.8 to 27.3)
		Percent	4.9 (1 to 9.9)	4.9 (1 to 10)	4.9 (1 to 9.8)	4.9 (1 to 9.9)	4.9 (1 to 9.9)	4.9 (1 to 9.9)	0.4 (-5.5 to 6.4)	0.2 (-6.1 to 7.1)	0.6 (-6.3 to 7.5)
	Secondhand smoke	Rate	17.1 (9.7 to 26.5)	9.4 (5.4 to 15.1)	24.2 (13.3 to 38.5)	18.4 (11 to 27.5)	14.4 (8.6 to 21.1)	22.6 (13.3 to 34.1)	7.8 (-16.4 to 39.1)	52.4 (0.2 to 91.1)	-6.5 (-30.1 to 29.2)
		Percent	6.3 (3.8 to 9.2)	7.9 (4.8 to 11.7)	5.8 (3.4 to 8.6)	6.5 (3.9 to 9.4)	8.2 (4.9 to 11.9)	5.8 (3.4 to 8.6)	3.8 (-6.5 to 15.9)	3 (-5.9 to 12.7)	-0.8 (-14.4 to 15.4)
	Smoking	Rate	163.2 (133.1 to 200.3)	21.7 (15.7 to 31.5)	295 (237.2 to 363.1)	148.4 (135.4 to 163.1)	31.3 (25.6 to 37.6)	266.8 (241.6 to 294.2)	-9.1 (-30.2 to 20.7)	44.2 (-7.2 to 110.6)	-9.6 (-31.3 to 20.3)
		Percent	59.6 (55.7 to 62.9)	18.2 (14.3 to 22.7)	70.7 (68.3 to 73.2)	52.2 (49.8 to 54.7)	17.7 (14.9 to 20.8)	67.9 (65.4 to 70.2)	-12.5 (-17.9 to -5)	-2.6 (-23.9 to 26.6)	-4.1 (-7.4 to -0.6)
YLDs	Diet low in fruits	Rate	0 (0 to 0.1)	0 (0 to 0)	0.1 (0 to 0.1)	0 (0 to 0.1)	0 (0 to 0)	0 (0 to 0.1)	-21.2 (-43.7 to 10.3)	16.9 (-24 to 70)	-33 (-56.9 to 1.3)
		Percent	1.7 (0.5 to 2.5)	1.9 (0.5 to 2.8)	1.6 (0.4 to 2.5)	1.2 (0.4 to 1.9)	1.3 (0.4 to 2.1)	1.1 (0.3 to 1.8)	-29.1 (-42.4 to -8.8)	-29.1 (-46.3 to -7)	-31.3 (-48.8 to -7.2)
	High fasting plasma glucose	Rate	0.1 (0 to 0.3)	0.1 (0 to 0.2)	0.2 (0 to 0.6)	0.3 (0.1 to 0.6)	0.2 (0 to 0.5)	0.4 (0.1 to 0.9)	99.9 (53.1 to 171.2)	230 (125 to 346.4)	67.5 (26.9 to 133)
		Percent	5.6 (1.2 to 12.8)	5.3 (1 to 12.7)	5.7 (0.9 to 13.8)	10.1 (2.5 to 21.4)	10.6 (2.1 to 23.8)	9.9 (1.7 to 22.6)	80.4 (62 to 122.2)	100.4 (85.3 to 126.2)	72.6 (61.6 to 91.2)
	Occupational carcinogens	Rate	0.1 (0.1 to 0.2)	0 (0 to 0)	0.2 (0.1 to 0.3)	0.1 (0.1 to 0.2)	0 (0 to 0)	0.3 (0.2 to 0.4)	18.2 (-12.8 to 56.6)	69.2 (11.9 to 139.2)	21.5 (-12.2 to 62.9)
		Percent	5 (3.3 to 6.9)	1.3 (0.9 to 1.8)	5.8 (3.9 to 8.1)	5.3 (3.6 to 7.1)	1.4 (1 to 1.8)	7.3 (5.1 to 9.8)	6.4 (-8.9 to 26.6)	2.5 (-14 to 24.6)	24.8 (6.6 to 47.1)
	Particulate matter pollution	Rate	0.6 (0.4 to 0.8)	0.3 (0.2 to 0.4)	0.9 (0.5 to 1.3)	0.6 (0.4 to 0.9)	0.4 (0.3 to 0.6)	0.8 (0.5 to 1.2)	6.9 (-16.6 to 36.6)	59.6 (5.6 to 100.4)	-6.4 (-28.6 to 23.8)
		Percent	22.8 (17.3 to 28.4)	22.7 (17.1 to 28.3)	22.9 (17.3 to 28.4)	22 (16.3 to 27.5)	22 (16.3 to 27.5)	22 (16.3 to 27.5)	-3.6 (-7.7 to -1.1)	-3.2 (-7.7 to -0.5)	-3.7 (-7.6 to -1.2)
	Residential radon	Rate	0.1 (0 to 0.3)	0.1 (0 to 0.1)	0.2 (0 to 0.4)	0.1 (0 to 0.3)	0.1 (0 to 0.2)	0.2 (0 to 0.4)	11.8 (-13.3 to 40.4)	66.5 (10.1 to 109.7)	-2.2 (-25.9 to 29.7)
		Percent	4.9 (1 to 9.8)	4.9 (1 to 9.9)	4.9 (1 to 9.8)	4.9 (1 to 9.8)	4.9 (1 to 10)	4.9 (1 to 9.8)	0.6 (-5.6 to 8.3)	0.6 (-6.1 to 8.8)	0.6 (-6.8 to 9)
	Secondhand smoke	Rate	0.2 (0.1 to 0.3)	0.1 (0 to 0.2)	0.2 (0.1 to 0.4)	0.2 (0.1 to 0.3)	0.1 (0.1 to 0.2)	0.2 (0.1 to 0.3)	15.2 (-10.4 to 48.8)	70.1 (13.8 to 117.8)	-3.8 (-28.8 to 33)
		Percent	6.3 (3.8 to 9.2)	7.6 (4.6 to 11.2)	5.9 (3.5 to 8.7)	6.5 (3.9 to 9.5)	7.9 (4.7 to 11.5)	5.8 (3.4 to 8.7)	3.8 (-6.6 to 15.9)	3.1 (-6.1 to 13.1)	-0.9 (-14.4 to 15.6)

Measure	Risk factor	Metric	Year						% Change (1990 to 2019)		
			1990			2019					
			Both	Female	Male	Both	Female	Male	Both	Female	Male
	Smoking	Rate	1.5 (1 to 2.1)	0.2 (0.1 to 0.3)	2.8 (1.8 to 3.8)	1.4 (1 to 1.9)	0.3 (0.2 to 0.5)	2.6 (1.8 to 3.3)	-4.3 (-26.7 to 25.4)	56.1 (-1.3 to 128.8)	-6.7 (-28.7 to 24.9)
		Percent	60.4 (56.3 to 63.9)	19.3 (15.3 to 24)	72.1 (69.5 to 74.5)	52.1 (49.4 to 54.7)	18.3 (15.3 to 21.6)	69.2 (66.7 to 71.6)	-13.8 (-19.4 to -6)	-5.3 (-26.4 to 22.3)	-4 (-7.3 to -0.6)

Data in parentheses are 95% Uncertainty Intervals (95% UIs)

DALYs=Disability-Adjusted Life Years; YLLs=Years of Life Lost; YLDs=Years Lived with Disability
